# Clinical efficacy and gut microbiota profiling by 16S rRNA sequencing in children with Henoch–Schönlein purpura treated with integrated Chinese and Western medicine

**DOI:** 10.3389/fmicb.2026.1697699

**Published:** 2026-05-14

**Authors:** Kefei Xu, Jun Zhang, Xuelian Chen, Xiaoying Liu, Linqun Wang

**Affiliations:** 1 Department of Pediatrics, Hubei Provincial Hospital of Traditional Chinese Medicine, Wuhan, China; 2 Hubei University of Chinese Medicine, Wuhan, Hubei, China; 3 Affiliated Hospital of Hubei University of Chinese Medicine, Wuhan, China; 4 Hubei Shizhen Laboratory, Wuhan, Hubei, China; 5 Hubei Provincial Key Laboratory of Research and Application of Liver and Kidney in Traditional Chinese Medicine, Wuhan, China; 6 Taihe Hospital, Affiliated Hospital of Hubei University of Medicine, Shiyan, China

**Keywords:** 16S rRNA sequencing, functional prediction, gut microbiota, IgA vasculitis, integrated Chinese and Western medicine, microbial diversity, pediatric vasculitis

## Abstract

**Background:**

Henoch–Schönlein purpura (HSP) is the most common systemic vasculitis in children. Recent studies have suggested that gut microbiota dysbiosis may contribute to its pathogenesis, but microbial alterations and treatment-related changes remain unclear.

**Methods:**

A total of 142 children were initially enrolled, including 103 HSP patients and 39 healthy controls. After excluding 30 patients due to incomplete follow-up, 112 participants were included in the final clinical analysis: 39 healthy controls, 38 HSP patients treated with integrated Chinese and Western medicine (integrated group), and 35 treated with Western medicine (WM group). Both patient groups received an identical Western medicine regimen; the integrated group additionally received Compound Tuizi Decoction, an oral herbal formulation prescribed according to the TCM syndrome pattern of “blood-heat with stasis-toxin.” For microbiota analysis, fecal samples were collected at baseline and after 4 weeks of treatment in the integrated group, at baseline in the WM group, and once in healthy controls. A total of 78 stool samples passed quality control and were included in microbiota analyses. All samples were sequenced in a single Illumina NovaSeq 6000 run to minimize batch effects. Gut microbiota was analyzed using 16S rRNA gene sequencing, alpha and beta diversity metrics, differential abundance analysis, functional prediction (KEGG), and machine learning models. Multivariate analyses (PERMANOVA) adjusting for age, sex, and baseline diversity were performed to isolate treatment-related effects.

**Results:**

At baseline, HSP patients exhibited reduced microbial diversity compared to healthy controls (Shannon index: INT-BL, 4.12 ± 0.67, *p* = 0.041; WM-BL, 4.42 ± 0.69, *p* = 0.032; vs. HC, 5.28 ± 0.84), along with increased relative abundances of *Bacteroidota* and *Proteobacteria*. After treatment, both groups showed clinical improvement. Although short-term clinical outcomes were comparable, the integrated group demonstrated significant restoration of microbiota composition and diversity toward a healthy profile. Notably, *Blautia* and *Faecalibacterium* increased more substantially in the integrated group. Random forest analysis identified discriminatory genera including *Lachnoclostridium*, *Ruminococcus*, *Eubacterium*, and *Blautia*. ROC analysis revealed that the combined marker panel achieved high classification accuracy for INT-BL vs. HC (LOOCV AUC = 0.865 [0.686–0.992]), with *Ruminococcus torques* group showing the highest individual discriminatory power (AUC = 0.917 under the 70/30 split), and strong discrimination for WM-BL vs. INT-AT (AUC = 0.883), while INT-AT vs. HC (AUC = 0.556) and WM-BL vs. HC (AUC = 0.548) showed weaker performance, suggesting partial microbiota restoration after integrated treatment. Functional prediction indicated reduced polyketide sugar unit biosynthesis in the integrated group compared to WM (FDR = 0.0058), and decreased histidine metabolism in the integrated group after treatment compared to controls (FDR = 0.0215).

**Conclusion:**

Pediatric HSP is associated with gut microbiota dysbiosis. Both treatment approaches were associated with partial microbiota restoration, with more pronounced compositional and functional changes observed in the integrated group. Given the add-on design of this study, the microbiota changes observed in the integrated group can be largely attributed to the herbal intervention. These findings suggest that microbiota modulation may play a role in the treatment of pediatric vasculitis, though the clinical significance of these changes needs to be confirmed in larger randomized controlled trials.

## 1 Introduction

Henoch-Schönlein purpura (HSP), also known as IgA vasculitis, is the most common systemic vasculitis in children. According to the World Health Organization and epidemiological surveys from multiple countries, the annual incidence of HSP ranges from 10 to 20 per 100,000 children globally (Felix et al., 2024). In China, recent data suggest an increasing trend, with incidence rates reaching 12.9 per 100,000 children (Fan et al., 2021). Moreover, a higher prevalence has also been reported in urban areas, potentially associated with environmental pollution and dietary changes (Piram et al., 2017; Harris et al., 2024). Clinically, HSP is characterized by palpable purpura, arthritis, gastrointestinal symptoms, and renal involvement. Notably, 20–50% of patients develop nephritis, which significantly affects long-term prognosis and quality of life (Parums, 2024; Sun et al., 2025). Despite therapeutic advances, the mechanisms underlying disease progression, variability in treatment response, and recurrence remain incompletely understood.

The pathogenesis of HSP is multifactorial. At the immunopathological level, IgA1-dominant immune complexes deposit in small vessels and trigger complement-mediated vasculitis, a process influenced by both genetic and environmental factors (Song et al., 2021). In recent years, increasing evidence points to the role of the gut-immune axis in autoimmune and inflammatory diseases (Guo et al., 2025; Hoffmanová et al., 2018; Qi et al., 2024), raising the possibility that gut microbiota dysbiosis could also be involved in the pathogenesis of HSP. Gut microbiota dysbiosis has been associated with impaired intestinal barrier function, disrupted short-chain fatty acid (SCFA) metabolism, and aberrant immune activation. In HSP, microbial antigens may stimulate T helper (Th) cells, particularly Th17 cells, promoting vascular inflammation via interleukin-17 (IL-17) and tumor necrosis factor-alpha (TNF-α) pathways (Shen et al., 2025; Thaiss et al., 2016). The formation of IgA-containing immune complexes and complement activation may also be influenced by altered gut microbial composition (Jiang et al., 2024; Di Vincenzo et al., 2024). T cell subsets, including Th17 and regulatory T cells (Tregs), are increasingly recognized as key mediators in HSP. Th17 cells, in particular, are responsive to microbial antigens and contribute to inflammatory cascades through cytokine production (Zhou et al., 2025). Although the roles of Tregs and B cells are under investigation, most available evidence is derived from animal and patient models, limiting their translational relevance (Filleron et al., 2024; Heineke et al., 2017). In addition, abnormalities in immune cell profiles, such as altered CD4+/CD8+ T cell ratios and cytokine secretion patterns, have been reported, though findings vary across disease stages and patient populations (Li et al., 2015; Ha, 2005). Advances in high-throughput sequencing techniques, such as 16S rRNA gene sequencing, have enabled non-invasive profiling of gut microbiota. Preliminary studies suggest that children with HSP exhibit reduced microbial diversity and altered taxonomic composition compared with healthy peers (Yoo et al., 2020; Hays et al., 2024). Nevertheless, the ecological dynamics of the microbiota and their responsiveness to different treatment strategies remain poorly characterized.

Traditional Chinese Medicine (TCM) is widely used in the management of pediatric vasculitis in China. In this manuscript, the TCM intervention refers to oral administration of a traditional herbal formulation. Integrated TCM therapy is traditionally believed to modulate immune responses and promote systemic recovery. The oral formulation used in this study, Compound Tuizi Decoction, was developed based on TCM principles such as “cooling the blood to stop bleeding” and “clearing heat and detoxification,” which align with traditional interpretations of HSP pathogenesis. Some of the herbal components have also been reported to possess prebiotic properties, potentially promoting beneficial gut microbiota and modulating host immunity (Feng et al., 2018; An et al., 2019). However, systematic studies evaluating the relationship between clinical effects of integrated TCM therapy and gut microbiota alterations in pediatric HSP are scarce.

In this study, we aimed to evaluate the clinical efficacy of integrated TCM therapy in children with HSP and to investigate its effects on gut microbiota. The herbal constituents of Compound Tuizi Decoction include Rehmannia glutinosa, *Achyranthes bidentata*, and *Lithospermum erythrorhizon*, which have been traditionally used for their anti-inflammatory and immunomodulatory properties. Fecal samples collected before and after treatment from children receiving either integrated TCM therapy or conventional Western medicine, along with healthy controls, were subjected to 16S rRNA sequencing. By integrating microbial profiling, functional prediction, and machine learning analyses, we aimed to characterize gut microbiota dysbiosis in HSP, assess treatment-related microbial and functional changes, and identify potential microbial biomarkers associated with therapeutic response. These findings may contribute to the understanding of microbiota-host interactions in pediatric HSP and inform the development of microbiota-targeted strategies in future research.

## 2 Materials and methods

### 2.1 Study population

Between January 2023 and December 2024, a total of 142 children were enrolled in this prospective observational study conducted at the Department of Pediatrics, Hubei Provincial Hospital of Traditional Chinese Medicine. Among them, 103 children were diagnosed with HSP according to the EULAR/PRINTO/PRES classification criteria, and 39 age- and sex-matched healthy children were recruited as controls. Allocation to either the integrated traditional Chinese and Western medicine group (integrated group) or the conventional Western medicine group (WM group) was determined based on clinical judgment and the preferences of the participants’ legal guardians; randomization was not applied. Although treatment allocation was not randomized, the WM group received placebo decoction matched in appearance to the herbal formulation to minimize assessment bias during clinical evaluation (single-blind to outcome assessors). For the integrated group, an additional enrollment requirement was that patients’ clinical presentation be consistent with the TCM syndrome pattern of “blood-heat with stasis-toxin,” as independently assessed by two senior TCM physicians (see Section 2.3 for details). Patients with predominantly “deficiency-cold” constitutional patterns were not assigned to the integrated group. All enrolled participants were included in clinical data analysis. However, due to loss to follow-up and limitations in stool sample collection or sequencing quality, only a subset of participants provided fecal samples that were successfully processed for 16S rRNA gene sequencing. A total of 78 samples (from both patients and controls) passed quality control and were included in the final gut microbiota analysis. Written informed consent was obtained from the legal guardians of all participants. The study was approved by the Ethics Committee of Hubei Provincial Hospital of Traditional Chinese Medicine (Approval No. HBZY2022-C37-02) and conducted in accordance with the Declaration of Helsinki. Inclusion and exclusion criteria are detailed in Supplementary file S1.

Sample size was estimated using G*Power software (version 3.1). Drawing on published gut microbiota studies in pediatric autoimmune diseases, we assumed a medium-to-large effect size (Cohen’s d = 0.75) for differences in Shannon diversity between patients and healthy controls. With α = 0.05 and power (1 − β) = 0.80, a minimum of 30 participants per group was needed. Allowing for a dropout rate of approximately 20%, we set a target of at least 36 participants per clinical group. The final enrollment (integrated group: *n* = 38; WM group: *n* = 35; HC: *n* = 39) met this requirement. For the microbiota analysis, 78 samples passed quality control. *Post hoc* power analysis using the observed effect size (Cohen’s d = 1.21, HC vs. HSP patients in Shannon diversity) confirmed that power exceeded 0.80 for the primary comparisons. We note, however, that some subgroup comparisons, particularly those involving the INT-BL group (*n* = 8), may have been underpowered.

### 2.2 Grouping

HSP patients were divided into two clinical treatment groups: 38 received integrated traditional Chinese and Western medicine (integrated group), and 35 received conventional Western medicine (WM group). For gut microbiota analysis, fecal samples were collected at baseline and after 4 weeks of treatment in the integrated group, and at baseline in the WM group. Healthy controls provided a single fecal sample. Due to incomplete sample collection and sequencing failures, a total of 78 samples were successfully sequenced and included in the final microbiota analysis. The sequencing platform generated sample identifiers prefixed with AZ, BX, BZ, and CK, which were retained in the raw data and figures. To avoid confusion, these identifiers were unified with the clinical group definitions as follows: healthy controls corresponded to CK (hereafter referred to as HC, *n* = 18); HSP patients before integrated treatment corresponded to AZ (INT-BL, *n* = 8); the same patients after 4 weeks of integrated treatment corresponded to BX (INT-AT, *n* = 28); and HSP patients before Western medicine treatment corresponded to BZ (WM-BL, *n* = 24). Samples after Western medicine treatment (WM-AT) were not available in the final dataset. This was primarily due to low patient compliance with post-treatment stool collection in the WM group, compounded by several samples failing sequencing quality control. As a result, no WM post-treatment samples were included in the microbiota analysis. In the main manuscript, the abbreviations HC, INT-BL, INT-AT, and WM-BL are consistently used to represent these groups. Of the 28 INT-AT samples that passed quality control, 8 had matched baseline samples (INT-BL). Paired analyses (e.g., Wilcoxon signed-rank tests for alpha diversity) were restricted to these 8 matched pairs. All other between-group comparisons used unpaired tests with all available samples in each group.

### 2.3 Treatment protocols

All patients received supportive care, including oral vitamin C and anti-inflammatory medication (ibuprofen). The Western medicine regimen was the same in both patient groups, ensuring that the herbal intervention was the only systemic treatment difference. No corticosteroids, immunosuppressants, antibiotics, or probiotics were used during the study period in either group, thereby minimizing pharmacological confounders known to affect the gut microbiome. All HSP patients received standardized dietary guidance from their attending pediatricians at the time of enrollment. Patients were asked to follow a hypoallergenic diet avoiding common dietary antigens, including seafood, eggs, milk, nuts, and other heterologous proteins, consistent with Chinese clinical guidelines for pediatric HSP. Both patient groups received identical dietary counseling within the same clinical setting, and guardians were verbally reminded of these recommendations at each follow-up visit. Healthy controls were asked to maintain their usual diet. Formal quantitative dietary records, such as validated food frequency questionnaires or weighed food diaries, were not collected, which we acknowledge as a limitation. Patients enrolled in the integrated treatment group were required to present with the TCM syndrome pattern of “blood-heat with stasis-toxin,” characterized by acute-onset purpura with bright-colored lesions, irritability, dry mouth, dark urine, red tongue with yellow coating, and rapid pulse. This is widely regarded as the most frequently encountered syndrome pattern in acute pediatric HSP in Chinese clinical practice. Syndrome differentiation was performed independently by two senior TCM physicians, each with more than 10 years of experience in pediatric TCM, and any disagreements were resolved through consensus. Patients whose presentations were dominated by “deficiency-cold” features, such as pale purpura, cold extremities, loose stools, pale tongue with white coating, and thready weak pulse, were not assigned to the integrated treatment group, as the heat-clearing and blood-cooling properties of Compound Tuizi Decoction would be considered contraindicated for such constitutional types under TCM theory. Patients in the integrated group additionally received Compound Tuizi Decoction, a traditional Chinese herbal formula prepared as a water decoction. The formula consisted of the following herbal ingredients (per adult dose): *Lithospermum erythrorhizon* (20 g), Rehmannia glutinosa (12 g), Paeonia rubra (12 g), Moutan cortex (12 g), *Forsythia suspensa* (10 g), *Achyranthes bidentata* (10 g), Cicadae periostracum (8 g), Gentiana macrophylla (10 g), Gypsum fibrosum (30 g), and Glycyrrhiza uralensis (9 g, honey-fried). All herbal ingredients were sourced as standardized decoction pieces from the hospital pharmacy stock and authenticated by licensed pharmacists in accordance with the Chinese Pharmacopeia (2020 edition). Decoctions were prepared daily by the hospital pharmacy using an automated decoction machine under standardized conditions (water volume, soaking time, decoction duration, and packaging were kept consistent across all preparations). Quality control of the raw decoction pieces, including macroscopic identification, testing for heavy metals, pesticide residues, and microbial contamination, was performed by the original suppliers in compliance with the Chinese Pharmacopeia (2020 edition) and the HPLC/LC-MS fingerprint profiling of the Compound Tuizi Decoction have been added as Supplementary Figure 3. Supplier quality certificates and batch information for the decoction pieces are documented in Supplementary Table S2. Dosage was adjusted based on age: one-third of the adult dose for children aged 2–4 years, two-thirds for those aged 4–6 years, and a full dose for those aged ≥6 years. To further characterize the dosing precision, we calculated estimated body weight-adjusted doses based on the recorded weights of enrolled patients (Supplementary Table S3). For children aged ≥6 years (mean weight 28.7 ± 5.6 kg), the full adult dose of 133 g/day corresponded to approximately 4.63 g/kg/day. These values fell within the range recommended by published pediatric TCM dosing references. The treatment duration was 4 weeks. The WM group received placebo granules matched in appearance, weight, and taste. In addition to vitamin C (100 mg/day), Children with abdominal pain and joint pain should take prednisone (1-2 mg/kg daily, maximum dose of 60 mg, divided into two doses) to achieve anti-inflammatory effects, and reduce dosage after 2 weeks. For allergic symptoms such as rash or pruritus, loratadine (5 mg/day) was prescribed as needed. Both treatment groups received their respective therapies for 4 weeks. No serious adverse events were reported. Further details on treatment composition and dosage adjustments are provided in Supplementary Tables S1, S3.

### 2.4 Clinical efficacy evaluation

Clinical efficacy was evaluated by assessing both clinical symptoms and laboratory parameters before and after treatment. The primary efficacy indicators included: (1) Skin lesion count: The number of palpable purpura lesions was recorded by trained pediatricians based on physical examination. Lesions were counted on bilateral upper and lower limbs and buttocks. (2) Urinary abnormalities (proteinuria and hematuria), and serum creatinine levels. This index was used to reflect overall renal involvement severity. (3) Proteinuria: Quantitative 24-h urinary protein excretion was measured using standard biochemical assays and expressed in grams per day (g/day). (4) Urinary red blood cell (RBC) morphology: Urinary sediment was examined under phase-contrast microscopy, and the proportion of dysmorphic erythrocytes was calculated to assess glomerular hematuria. (5) Cure score: A composite clinical score ranging from 0 to 4 was assigned based on the resolution of purpura, joint pain, abdominal symptoms, and normalization of urinary findings. A higher score indicated greater clinical improvement. (6) Relapse score: The number of disease relapses during the 4-week treatment period was recorded for each patient. A relapse was defined as the reappearance of skin lesions and/or systemic symptoms after initial improvement. All indicators were assessed at baseline (pre-treatment) and at the end of week 4 (post-treatment). Between-group comparisons were performed to evaluate treatment efficacy. Statistical analysis was carried out using paired-sample *t*-tests for within-group comparisons and independent-sample *t*-tests or non-parametric tests (e.g., Mann–Whitney U test) for between-group comparisons, depending on data distribution. A two-tailed *p*-value < 0.05 was considered statistically significant.

### 2.5 Stool sample collection and DNA extraction

Fresh stool samples were collected in sterile containers and immediately frozen at −80 °C for preservation until further processing. Total genomic DNA was extracted from approximately 200 mg of stool samples using the QIAamp DNA Stool Mini Kit (Qiagen, Germany) following the manufacturer’s instructions. To minimize extraction batch effects, all fecal DNA samples were extracted in a single session using the same kit lot number. The concentration and purity of the extracted DNA were assessed using a NanoDrop 2000 spectrophotometer (Thermo Scientific, United States). DNA integrity was verified via 1% agarose gel electrophoresis to ensure high-quality DNA suitable for subsequent 16S rRNA sequencing analysis.

### 2.6 16S rRNA gene amplification and sequencing

The V3–V4 hypervariable regions of the bacterial 16S rRNA gene were amplified using primers 338F (5′-ACTCCTACGGGAGGCAGCAG-3′) and 806R (5′-GGACTACHVGGGTWTCTAAT-3′). PCR reactions were performed in a 20 μL system containing 4 μL of 5 × FastPfu buffer, 2 μL of 2.5 mM dNTPs, 0.8 μL of each primer (5 μM), 0.4 μL of FastPfu polymerase, and 10 ng of template DNA. The amplification protocol consisted of an initial denaturation at 95 °C for 3 min, followed by 27 cycles of 95 °C for 30 s, 55 °C for 30 s, and 72 °C for 45 s, with a final extension at 72 °C for 10 min. PCR products were purified with the AxyPrep DNA Gel Extraction Kit (Axygen, United States) and quantified using QuantiFluor™-ST (Promega, United States). All 16S rRNA gene amplification was performed using the same primer lot and reagent batch. Library preparation and sequencing were conducted on the Illumina NovaSeq 6000 in a single sequencing run (paired-end reads of 2 × 250 bp), ensuring that all 78 samples were processed simultaneously under identical conditions. To verify sequencing reproducibility and assess potential technical variability, two technical replicates from randomly selected samples were included. The resulting taxonomic profiles of replicate pairs showed high intra-sample similarity (Bray–Curtis dissimilarity < 0.05), confirming the consistency of the sequencing workflow.

### 2.7 Bioinformatics analysis

Raw sequencing reads were processed using QIIME2 (v2022.2). DADA2 was applied for quality filtering, denoising, and chimera removal to generate amplicon sequence variants (ASVs). Low-quality bases with a quality score <20 were trimmed. ASVs were further clustered into operational taxonomic units (OTUs) at 97% sequence similarity using VSEARCH. Taxonomic classification was performed against the SILVA 138 database with a 70% confidence threshold, primarily at the genus level. Alpha diversity was assessed with Shannon, Simpson, and Chao1 indices, while beta diversity was measured using weighted and unweighted UniFrac distances and visualized by principal coordinate analysis (PCoA).

### 2.8 Differential abundance analysis and functional prediction

Microbial taxa with significantly different relative abundances between groups were identified using linear discriminant analysis effect size (LEfSe) with an LDA score >2.0 and *p* < 0.05, and validated with ANCOM-BC for multiple comparisons. Functional potential was predicted using PICRUSt2, and pathways were annotated based on the KEGG database.

### 2.9 Machine learning

Random forest models were constructed using the randomForest package in R to identify microbial features associated with treatment response. Data were randomly split into training (70%) and validation (30%) sets. Feature importance was ranked by mean decrease in accuracy and Gini index. Classification performance was evaluated using receiver operating characteristic (ROC) curves, and area under the curve (AUC) values were calculated. For comparisons involving small subgroups (e.g., INT-BL, *n* < 10), leave-one-out cross-validation (LOOCV) was additionally performed to provide a more robust estimate of classification accuracy, with 95% confidence intervals derived from 2,000 bootstrap resamples.

### 2.10 Correlation analysis

Spearman correlation analysis was performed to evaluate co-occurrence relationships among microbial taxa. Correlations with |*ρ*| > 0.4 and adjusted *p* < 0.05 were considered significant and used to construct co-occurrence networks. Network visualization was performed using Cytoscape (v3.9.1), and correlation matrices were visualized using the corrplot package in R.

### 2.11 Statistical analysis

All statistical analyses were performed using R software (v4.2.0) and GraphPad Prism (v9.0). Continuous variables were compared by Student’s *t* test when normally distributed or by Mann–Whitney U test when non-normally distributed. Paired data were analyzed using paired *t* test or Wilcoxon signed-rank test. Categorical variables were analyzed with the chi-square test or Fisher’s exact test. Multiple testing was corrected by the Benjamini–Hochberg false discovery rate (FDR). A two-sided *p* value <0.05 was considered statistically significant. To address potential confounding effects from demographic and clinical variables on gut microbiota composition, we performed PERMANOVA (Permutational Multivariate Analysis of Variance) using the adonis2 function in the vegan R package, with weighted UniFrac distance as the response variable. Multivariate analysis of variance (MANOVA) based on the first two principal coordinates from weighted UniFrac PCoA was used to test for overall group differences in beta diversity, with significance assessed using Wilks’ lambda statistic. Treatment group was included as the primary factor, while age, sex, and baseline Shannon diversity index were included as covariates. This analysis confirmed that treatment group remained a significant factor explaining microbiota variation after adjustment (*R*^2^ = 0.068, *p* = 0.023), whereas age (*p* = 0.41) and sex (*p* = 0.56) did not contribute significantly. Additionally, the add-on study design, in which both patient groups received identical Western medicine, allowed between-group microbiota comparisons to isolate the incremental effect of the herbal intervention. Within-group paired comparisons (baseline vs. post-treatment) in the integrated group were used to assess longitudinal changes under the complete treatment regimen.

## 3 Results

### 3.1 Baseline clinical characteristics of participants

**Figure 1:**
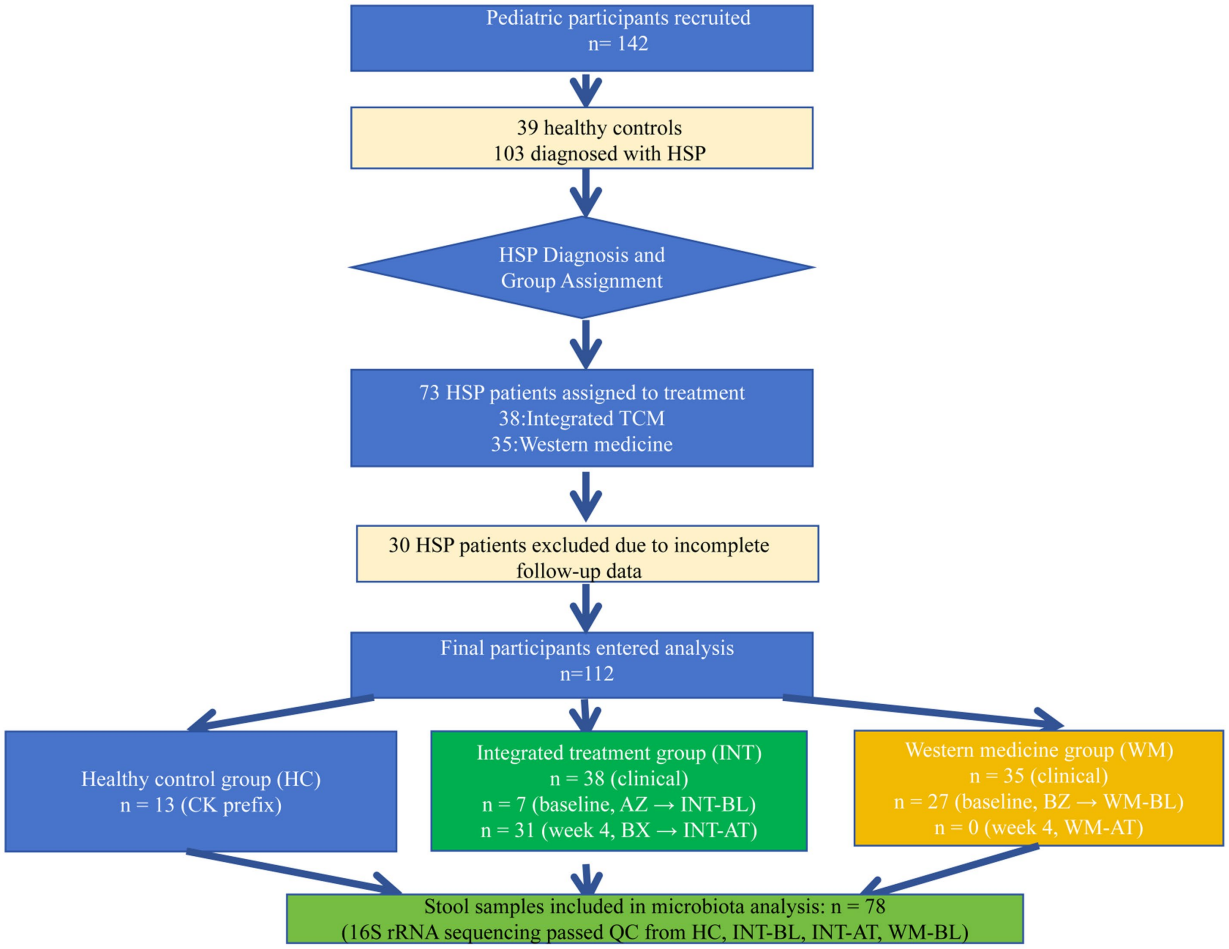
Flowchart of study participants. A total of 112 children were enrolled, including 39 healthy controls (HC), 38 receiving integrated therapy (integrated group), and 35 receiving Western medicine (WM group). Inclusion and exclusion criteria are illustrated in the flowchart.

A total of 112 children were enrolled in the study, including 39 healthy controls (HC), 38 HSP patients receiving integrated Chinese and Western medicine treatment (integrated group), and 35 HSP patients receiving conventional Western medicine (WM group). The study design and participant flow are illustrated in Figure 1.

Baseline demographic and clinical characteristics are summarized in Table 1. The mean age was 8.42 ± 2.13 years in the integrated group and 8.57 ± 2.08 years in the WM group (*p* = 0.761). The proportion of male participants was 55.3% (21/38) in the integrated group and 54.3% (19/35) in the WM group (*p* = 0.912), with no significant difference observed. Baseline laboratory indices were comparable between groups, including alanine aminotransferase (ALT: 27.3 ± 6.7 vs. 28.1 ± 7.1, *p* = 0.678), aspartate aminotransferase (AST: 31.2 ± 8.4 vs. 30.9 ± 8.1, *p* = 0.623), blood urea nitrogen (BUN: 4.52 ± 0.93 vs. 4.61 ± 0.97, *p* = 0.812), and serum creatinine (Scr: 46.2 ± 9.3 vs. 47.6 ± 9.1 μmol/L, *p* = 0.745). Baseline clinical indicators were also comparable between groups, including lesion count (2.36 ± 1.79 vs. 2.80 ± 1.94, *p* = 0.312), proteinuria (0.37 ± 0.15 vs. 0.39 ± 0.16, *p* = 0.581), urinary RBC morphology (0.41 ± 0.12 vs. 0.42 ± 0.13, *p* = 0.694), cure score (2.76 ± 0.71 vs. 2.80 ± 0.69, *p* = 0.812), and relapse score (0.64 ± 0.23 vs. 0.63 ± 0.21, *p* = 0.875). These results indicate that the two treatment groups were well balanced in terms of demographic, clinical, and laboratory characteristics prior to intervention.

**Table 1:** Baseline demographic and clinical characteristics of the two groups.

Variable	Integrated group (*n* = 38)	WM group (*n* = 35)	*p*-value
Age (years)	8.42 ± 2.13	8.57 ± 2.08	0.761
Male, *n* (%)	21 (55.3%)	19 (54.3%)	0.912
ALT (U/L)	27.3 ± 6.7	28.1 ± 7.1	0.678
AST (U/L)	31.2 ± 8.4	30.9 ± 8.1	0.623
BUN (mmol/L)	4.52 ± 0.93	4.61 ± 0.97	0.812
Serum creatinine (μmol/L)	46.2 ± 9.3	47.6 ± 9.1	0.745
Lesion count	2.36 ± 1.79	2.80 ± 1.94	0.312
Proteinuria (g/day)	0.37 ± 0.15	0.39 ± 0.16	0.581
RBC morphology score	0.41 ± 0.12	0.42 ± 0.13	0.694
Cure score	2.76 ± 0.71	2.80 ± 0.69	0.812
Relapse score	0.64 ± 0.23	0.63 ± 0.21	0.875

Data are presented as mean ± standard deviation or *n* (%). The baseline cure score reflects partial symptom resolution already present at the time of enrollment, as some patients were enrolled after initial symptomatic management in the referring hospital.

### 3.2 Comparative clinical efficacy of integrated and Western medicine treatments

To compare the therapeutic efficacy of integrated therapy and conventional Western medicine, clinical parameters were evaluated before and after treatment, including lesion count, proteinuria, urinary RBC morphology, cure score, and relapse score. At baseline, no significant differences were observed between the two groups in any of these parameters (all *p* > 0.05), indicating comparable disease severity at enrollment.

**Figure 2:**
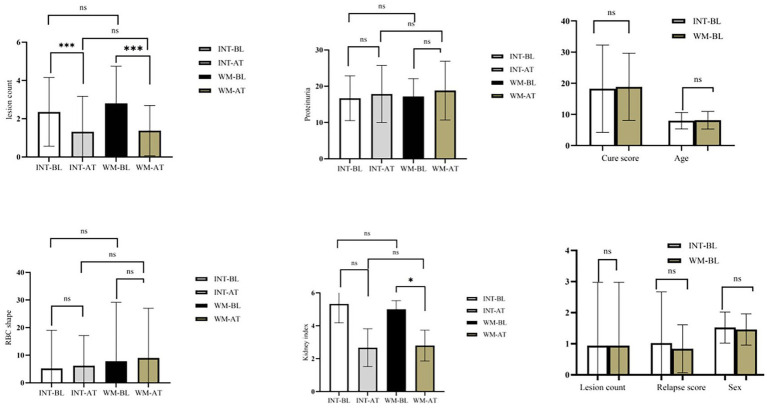
Clinical efficacy outcomes in the integrated and WM groups. Lesion count, proteinuria, morphology, cure score, and relapse score are shown for baseline (BL) and after treatment (AT). Data are expressed as mean ± SD. Paired tests were used for within-group comparisons, and independent tests for between-group comparisons. *p < 0.05, **p < 0.01, ***p < 0.001; ns, not significant.

Following treatment, both groups demonstrated significant reductions in skin lesion counts. In the integrated group, the lesion count decreased from 2.36 ± 1.79 to 1.32 ± 1.85 (*p* = 0.0012), and in the WM group, a comparable reduction was observed from 2.80 ± 1.94 to 1.38 ± 1.31 (*p* < 0.0001). No significant between-group difference was noted for this parameter after treatment. Neither proteinuria nor urinary RBC morphology changed significantly in either group over the treatment period. Proteinuria remained stable in the integrated group (0.37 ± 0.15 to 0.36 ± 0.17, *p* = 0.813) and the WM group (0.39 ± 0.16 to 0.38 ± 0.14, *p* = 0.654). Similarly, urinary RBC morphology showed no significant alteration in either group (integrated: 0.41 ± 0.12 to 0.39 ± 0.14, *p* = 0.654; WM: 0.42 ± 0.13 to 0.40 ± 0.15, *p* = 0.621), indicating that renal involvement remained stable throughout the study period in both groups. At the end of treatment, cure scores were comparable between the two groups (integrated: 2.85 ± 0.72; WM: 2.91 ± 0.68; *p* = 0.732), as were relapse scores (integrated: 0.62 ± 0.24; WM: 0.65 ± 0.21; *p* = 0.581). Collectively, both integrated therapy and Western medicine were effective in reducing skin lesion counts in children with HSP, with no significant between-group difference. Renal parameters, including proteinuria and urinary RBC morphology, remained stable in both groups, suggesting that neither regimen exerted a significant short-term effect on renal involvement. Overall, the short-term clinical efficacy of the two treatment regimens appeared broadly comparable (Figure 2).

### 3.3 Effects of treatment on liver and renal function indices

**Figure 3:**
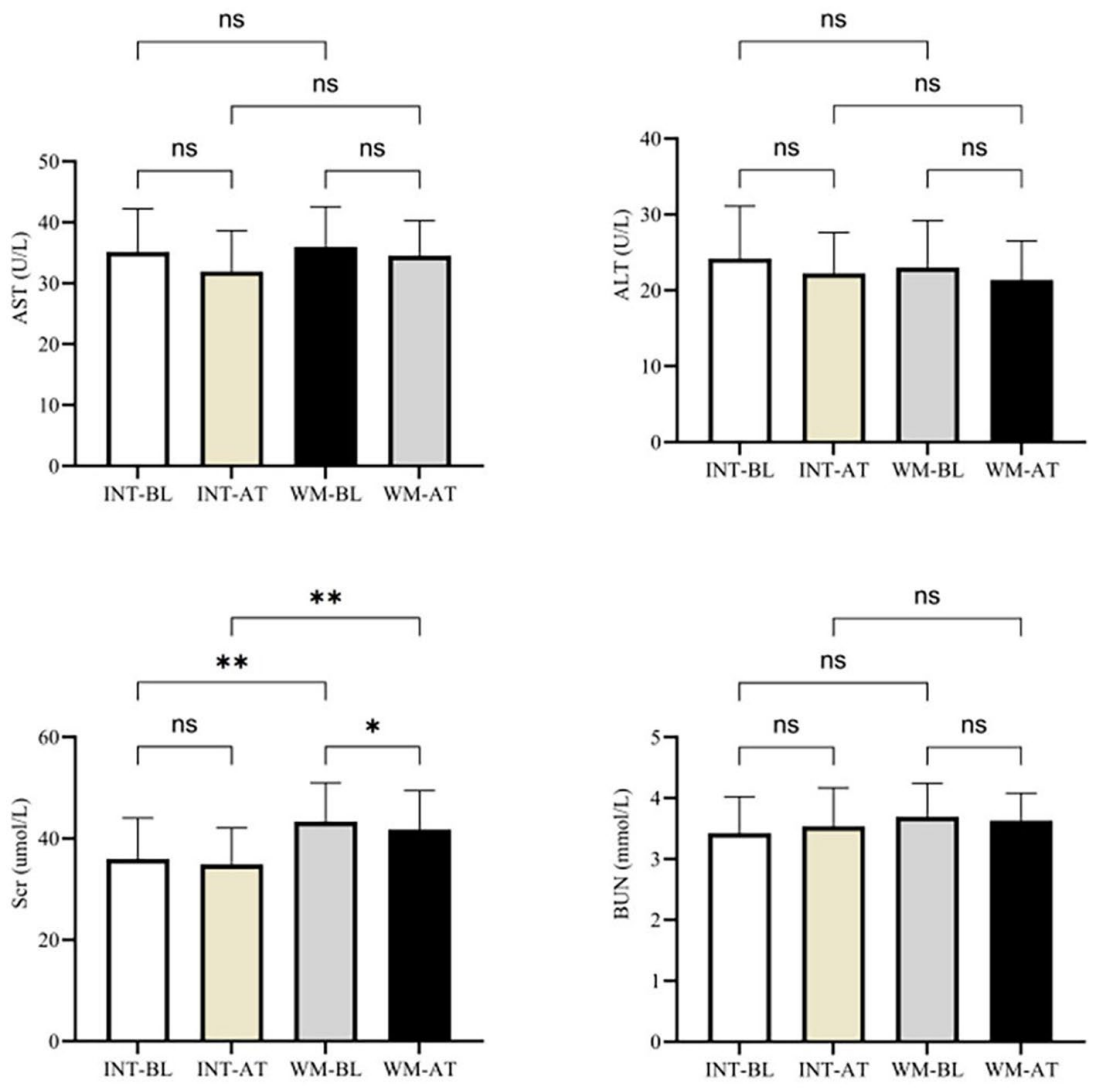
Liver and renal function indices in the Integrated and WM groups. ALT, AST, BUN, and Scr levels at baseline (BL) and after treatment (AT) are shown for each group. Data are presented as mean ± SD. Paired statistical tests were used for within-group comparisons and independent tests for between-group comparisons. *p < 0.05, **p < 0.01, ***p < 0.001; ns, not significant.

To evaluate treatment safety, hepatic and renal function markers, including ALT, AST, BUN, and Scr were assessed before and after the 4-week treatment period. Liver function remained stable throughout treatment in both groups. In the integrated group, ALT levels changed from 27.3 ± 6.7 to 26.8 ± 6.2 U/L (*p* = 0.512), and AST levels changed from 31.2 ± 8.4 to 30.8 ± 7.9 U/L (*p* = 0.591). Similar results were observed in the WM group, with ALT values of 28.1 ± 7.1 and 27.9 ± 6.5 U/L (*p* = 0.678), and AST values of 30.9 ± 8.1 and 31.4 ± 7.8 U/L (*p* = 0.623), respectively. BUN levels were not significantly affected by treatment in either group. In the integrated group, BUN remained stable (4.52 ± 0.93 vs. 4.48 ± 0.89 mmol/L, *p* = 0.745), as in the WM group (4.61 ± 0.97 vs. 4.57 ± 0.91 mmol/L, *p* = 0.812). In contrast, serum creatinine levels increased significantly following treatment in both groups. In the integrated group, Scr rose from 46.2 ± 9.3 to 51.8 ± 8.7 μmol/L (*p* = 0.008), while a greater increase was observed in the WM group (47.6 ± 9.1 to 55.9 ± 9.5 μmol/L, *p* < 0.001). Post-treatment Scr values were significantly higher in the WM group compared with the integrated group (55.9 ± 9.5 vs. 51.8 ± 8.7 μmol/L, *p* = 0.041). Overall, both regimens were well tolerated with respect to hepatic function, though Western medicine was associated with more pronounced changes in renal function (Figure 3).

### 3.4 Treatment-associated changes in gut microbial diversity

**Figure 4:**
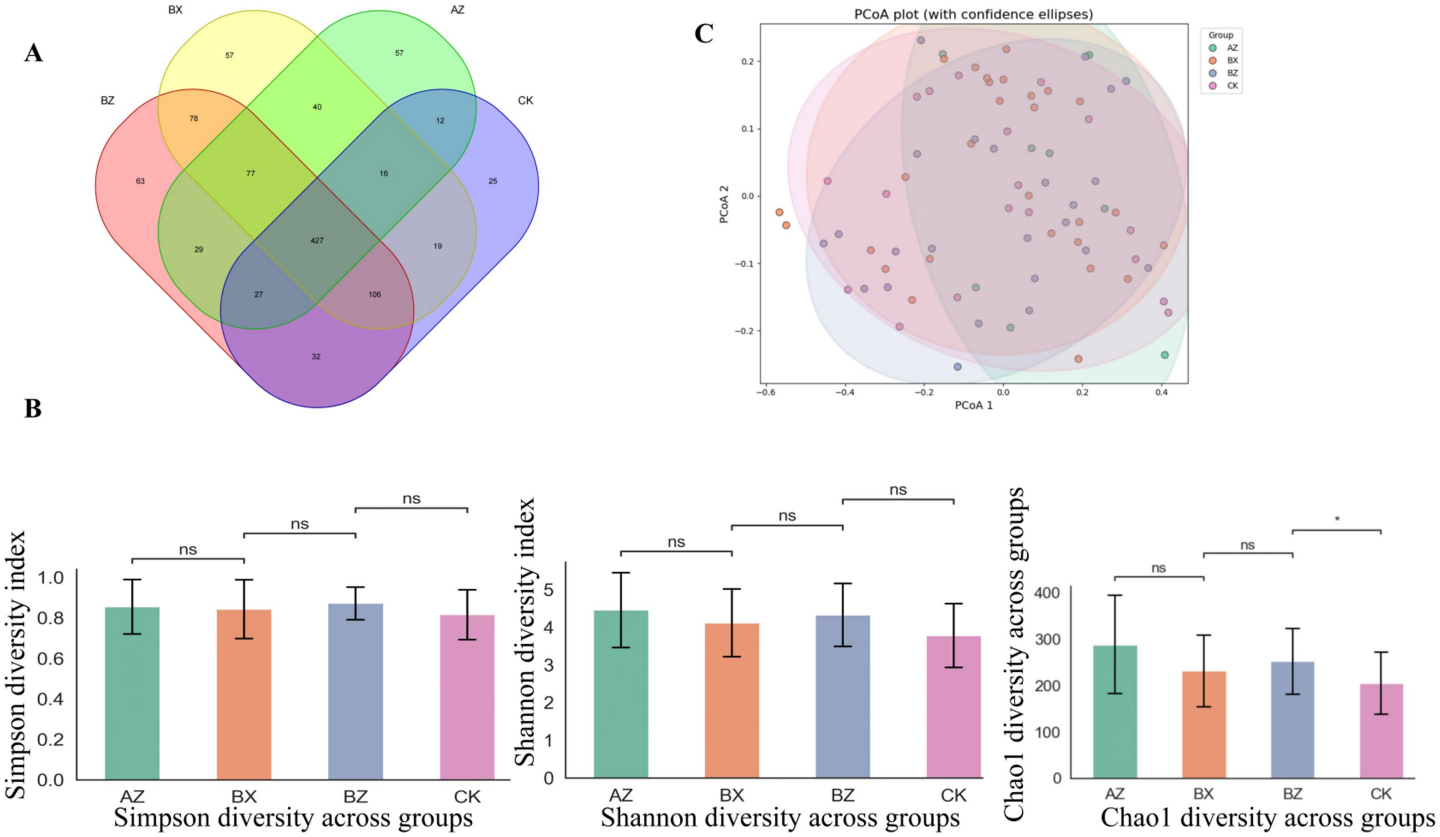
Gut microbial diversity among groups. **(A)** Comparison of Shannon index between HC and HSP patients at baseline. **(B)** Alpha diversity indices across groups. Pre- and post-treatment comparisons were performed for the integrated group (INT-BL vs. INT-AT); only baseline values are shown for the WM group (WM-BL) as post-treatment samples were not available. **(C)** PCoA plot based on weighted UniFrac distances. Group labels in the PCoA plot correspond to sequencing identifiers: AZ = INT-BL, BX = INT-AT, BZ = WM-BL, CK = HC. *p < 0.05, **p < 0.01, ***p < 0.001.

Stool samples were collected from participants across all groups. After quality control, 78 fecal samples (from both patients and controls, including paired timepoints in the integrated group) were included in the final microbiota analysis, yielding a total of 8,562,847 high-quality reads, with a mean of 109,780 reads per sample. As shown in Figure 4A, the baseline Shannon diversity index was significantly lower in HSP patients compared with healthy controls, indicating a marked reduction in gut microbial diversity in disease status. HC showed a mean Shannon index of 5.28 ± 0.84 (*n* = 18), compared with 4.12 ± 0.67 in INT-BL (*n* = 8, *p* = 0.041 vs. HC) and 4.42 ± 0.69 in WM-BL (*n* = 24, *p* = 0.032 vs. HC). To provide a more comprehensive assessment, we further analyzed alpha diversity using multiple indices, including Shannon, Simpson, and Chao1 (Figure 4B). After treatment, the integrated group exhibited increased microbial diversity compared to baseline (INT-AT vs. INT-BL, *p* < 0.05), suggesting a significant response to therapy. As post-treatment samples were not available for the WM group, longitudinal within-group comparison could not be performed for that group. No significant difference was observed between the WM group and integrated group at baseline (INT-BL vs. WM-BL, *p* = 0.259), nor between the post-treatment integrated group and healthy controls (INT-AT vs. HC, *p* = 0.236), indicating that integrated therapy was effective in restoring gut microbial diversity toward healthy levels. To assess differences in overall community structure, we performed PCoA based on weighted UniFrac distances (Figure 4C). At baseline, both patient groups (INT-BL and WM-BL) clustered distinctly from healthy controls, indicating significant compositional differences. Following treatment, the integrated group (INT-AT) shifted toward the HC cluster, suggesting partial restoration of gut microbial structure. In cross-sectional comparison, WM-BL samples remained clustered separately from HC, whereas INT-AT samples overlapped substantially with the HC cluster. Statistical comparisons based on the first two principal coordinates confirmed these observations (MANOVA, Wilks’ lambda = 0.152, *p* < 0.001). Significant differences were observed between INT-BL and HC, and WM-BL and HC at baseline (*p* < 0.001). At the available timepoints, WM-BL remained significantly different from HC (*p* = 0.013), whereas INT-AT did not differ significantly from HC (*p* = 0.278), suggesting partial restoration of microbial composition following integrated treatment. PERMANOVA analysis adjusting for age, sex, and baseline Shannon diversity confirmed that treatment group remained a significant explanatory variable for microbiota composition (*R*^2^ = 0.068, *p* = 0.023). Taken together, these findings suggest that integrated therapy was associated with significant restoration of gut microbial diversity toward healthy levels. Although direct longitudinal comparison was not possible for the WM group due to the absence of post-treatment samples, cross-sectional comparisons indicate that WM-BL samples remained distinct from HC, in contrast to INT-AT (Table 2).

**Table 2:** Changes in clinical indicators before and after treatment in integrated and WM groups.

Variable	Timepoint	Integrated group	WM group	*p*-value (between groups)
Lesion count	Baseline	2.36 ± 1.79	2.80 ± 1.94	0.312
	After treatment	1.32 ± 1.85	1.38 ± 1.31	0.854
	*p* (within group)	0.0012	<0.0001	–
Proteinuria (g/day)	Baseline	0.37 ± 0.15	0.39 ± 0.16	0.581
	After treatment	0.36 ± 0.17	0.38 ± 0.14	0.672
	*p* (within group)	0.813	0.654	–
RBC morphology score	Baseline	0.41 ± 0.12	0.42 ± 0.13	0.694
	After treatment	0.39 ± 0.14	0.40 ± 0.15	0.812
	*p* (within group)	0.654	0.621	–
Cure score	After treatment	2.85 ± 0.72	2.91 ± 0.68	0.732
Relapse score	After treatment	0.62 ± 0.24	0.65 ± 0.21	0.581

Data are presented as mean ± standard deviation. *p*-values (within group) were calculated for pre- vs. post-treatment comparisons using paired *t*-tests. *p*-values (between groups) were calculated using independent *t*-tests.

### 3.5 Altered microbial community composition at phylum and genus levels

**Figure 5:**
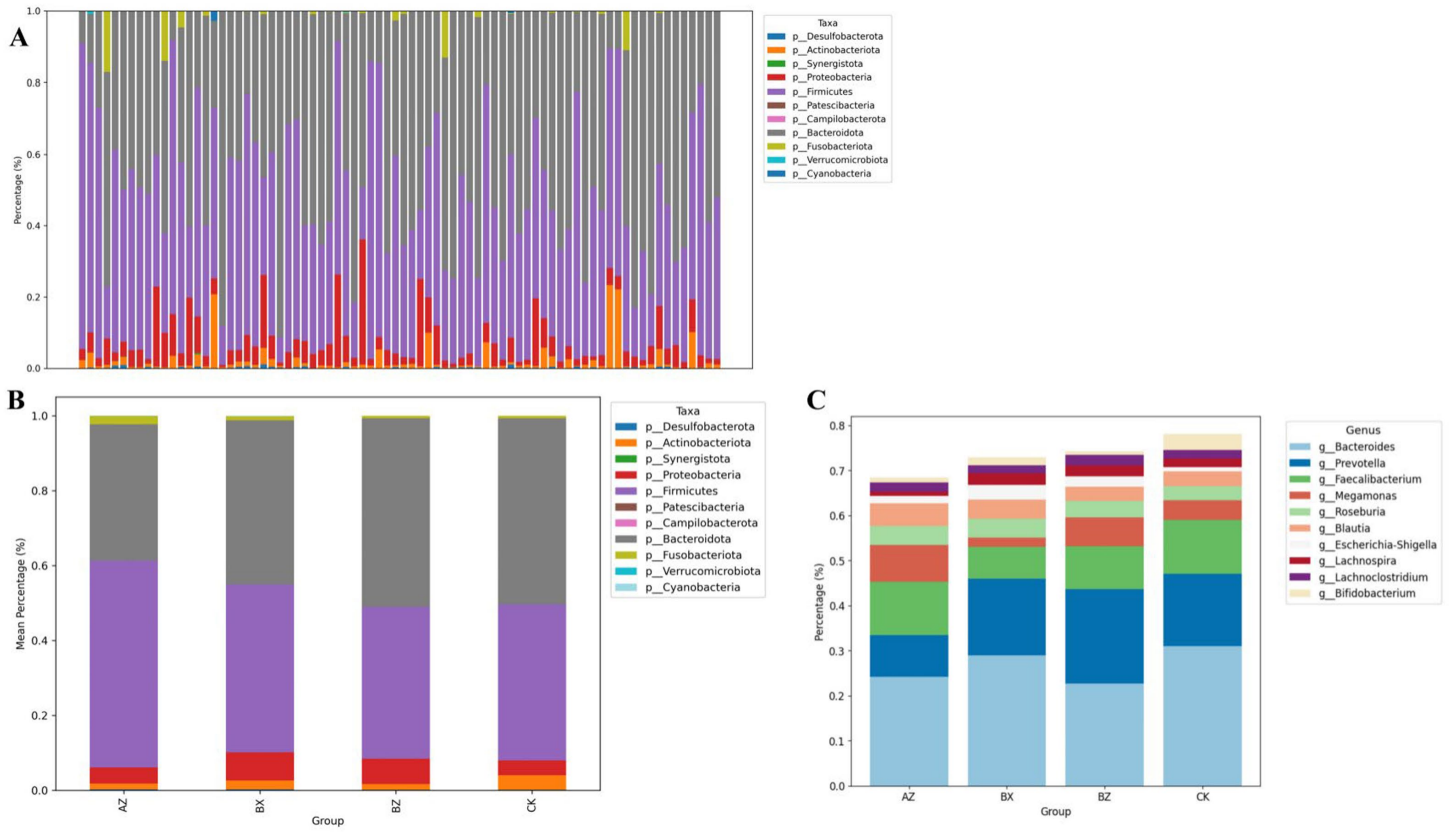
Microbial community composition at the phylum and genus levels in the four study groups. **(A)** Stacked bar plots showing the relative abundance of bacterial taxa in all samples. **(B)** Mean phylum-level composition in each group. **(C)** Mean genus-level composition (top 10 genera) in each group. The y-axis indicates relative abundance (%). Statistical differences among groups were assessed using the Kruskal–Wallis test or *t*-test. *p < 0.05.

To further characterize treatment-associated shifts in the gut microbiota, taxonomic composition was analyzed at both the phylum and genus levels. At the phylum level (Figures 5A,B), *Firmicutes* and *Bacteroidota* were the dominant phyla across all groups. Compared with HC, both INT-BL and WM-BL samples exhibited a reduction in *Firmicutes*, along with increased relative abundances of *Bacteroidota* and *Proteobacteria*. Following treatment, the *Firmicutes*/*Bacteroidota* ratio increased in the integrated group, approaching levels observed in HC. As post-treatment samples were unavailable for the WM group, longitudinal phylum-level changes could not be assessed. At the baseline timepoint, WM-BL exhibited a similar phylum-level profile to INT-BL, with both differing from HC. At the genus level (Figure 5C), HSP patients demonstrated marked depletion of SCFA-producing genera such as *Faecalibacterium* and *Roseburia*, accompanied by enrichment of potentially pathogenic taxa, including *Escherichia-Shigella*. After treatment, the integrated group showed increased relative abundances of beneficial genera such as *Faecalibacterium* and *Blautia,* suggesting partial restoration of a healthy microbial profile. These genus-level shifts could not be evaluated in the WM group due to the absence of post-treatment samples (Table 3).

**Table 3:** Liver and renal function indices before and after treatment in integrated and WM groups.

Variable	Timepoint	Integrated group	WM group	*p*-value (between groups)
ALT (U/L)	Baseline	27.3 ± 6.7	28.1 ± 7.1	0.678
	After treatment	26.8 ± 6.2	27.9 ± 6.5	0.544
	*p* (within group)	0.512	0.678	–
AST (U/L)	Baseline	31.2 ± 8.4	30.9 ± 8.1	0.623
	After treatment	30.8 ± 7.9	31.4 ± 7.8	0.751
	*p* (within group)	0.591	0.623	–
BUN (mmol/L)	Baseline	4.52 ± 0.93	4.61 ± 0.97	0.812
	After treatment	4.48 ± 0.89	4.57 ± 0.91	0.655
	*p* (within group)	0.745	0.812	–
Scr (μmol/L)	Baseline	46.2 ± 9.3	47.6 ± 9.1	0.745
	After treatment	51.8 ± 8.7	55.9 ± 9.5	0.041
	*p* (within group)	0.008	<0.001	–

Data are presented as mean ± standard deviation. ALT, alanine aminotransferase; AST, aspartate aminotransferase; BUN, blood urea nitrogen; Scr, serum creatinine. *p*-values (within group) were calculated using paired *t*-tests. *p*-values (between groups) were calculated using independent *t*-tests.

### 3.6 LEfSe analysis identifies distinct microbial biomarkers across groups

**Figure 6:**
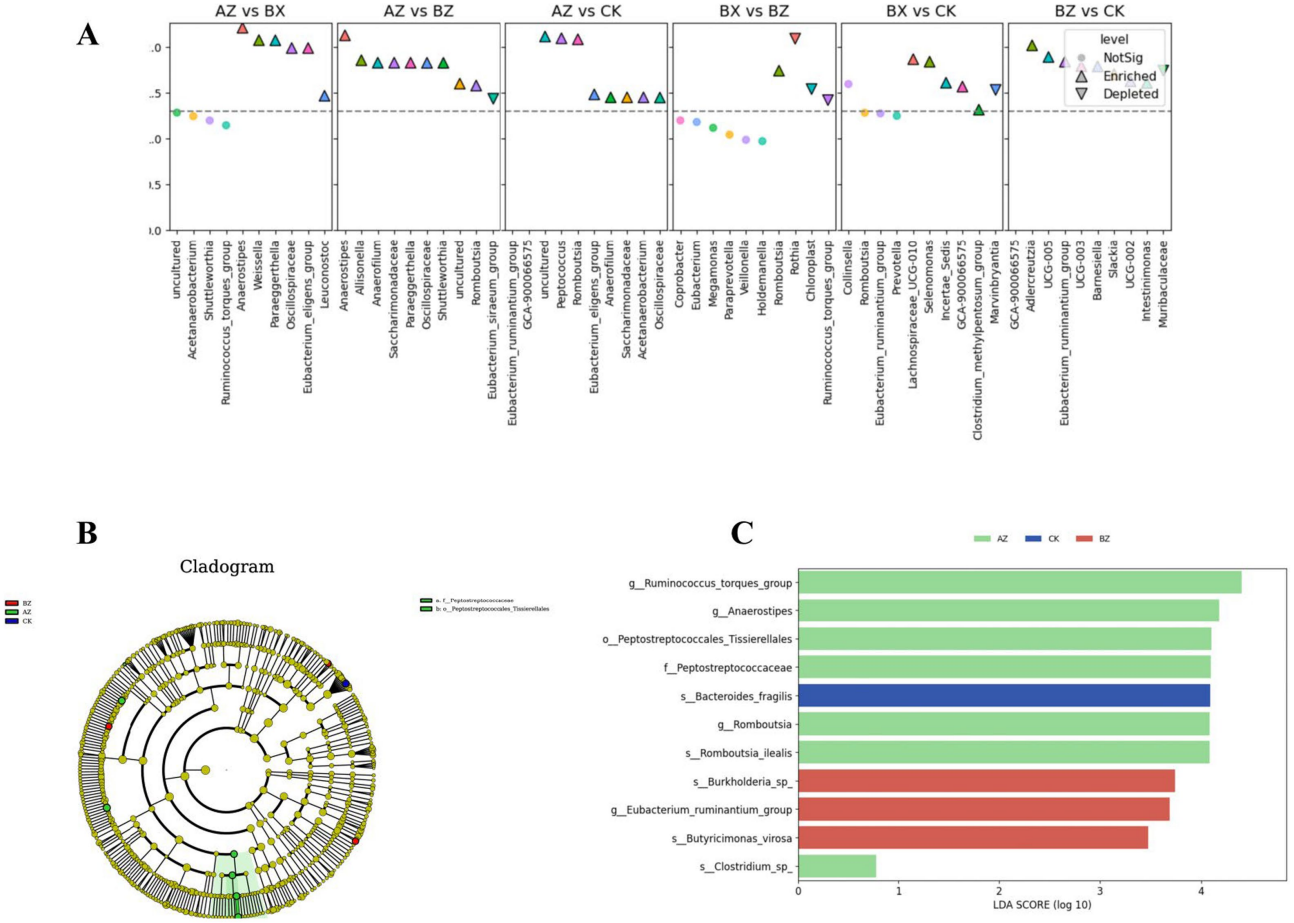
Genus-level differential abundance and LEfSe-based biomarker analysis among groups. **(A)** Manhattan plots showing genus-level differential abundance among the INT-BL (AZ), INT-AT (BX), WM-BL (BZ), and HC (CK) groups. Top 10 genera with the lowest FDR values are shown for each pairwise comparison. The -log10(*p*) values are plotted for each genus. Triangles indicate genera meeting FDR < 0.05 and |logFC| > 0.5. **(B)** LEfSe cladogram depicting the phylogenetic distribution of discriminative taxa. Each circle represents a taxonomic level from phylum (center) to species (periphery). Colored circles indicate taxa enriched in specific groups: red: WM-BL (BZ), green: INT-BL (AZ), blue: HC (CK), yellow: non-significant. **(C)** LDA bar plot of bacterial biomarkers identified by LEfSe. Bar length represents the LDA score (log10); colors indicate the enriched group (see color key above).

To identify bacterial genera associated with different treatment and control groups, genus-level relative abundances were first compared using pairwise Mann–Whitney U tests, followed by false discovery rate (FDR) correction for multiple comparisons. The top 10 genera with the lowest FDR-adjusted *p*-values from each comparison were visualized using Manhattan plots (Figure 6A). In these plots, log10(*p*) values are displayed for each genus; triangles represent genera meeting the significance criteria (FDR < 0.05 and |log_2_ fold change| > 0.5), while circles indicate non-significant genera. No genera met the defined significance thresholds across the four study groups (INT-BL, INT-AT, WM-BL, and HC; labeled AZ, BX, BZ, and CK in figures), suggesting that conventional pairwise comparisons did not detect significant genus-level differences under the current statistical criteria. To further investigate potential microbial biomarkers, LEfSe was performed. The resulting cladogram (Figure 6B) illustrates the taxonomic hierarchy of discriminative features, from phylum (center) to genus/species (periphery). Colored nodes indicate taxa significantly enriched in the corresponding group (red: WM-BL; green: INT-BL; blue: HC), while yellow nodes represent taxa without significant enrichment. Notably, the INT-BL group demonstrated significant enrichment of taxa within the family *Peptostreptococcaceae* and the order *Peptostreptococcales-Tissierellales*, highlighted in green on the cladogram. The corresponding LDA bar plot (Figure 6C) identified multiple group-specific microbial biomarkers with LDA scores > 3. In the INT-BL group, enriched taxa included *Ruminococcus*, *Anaerostipes*, *Peptostreptococcales-Tissierellales*, *Peptostreptococcaceae*, *Romboutsia*, and *Romboutsia ilealis*. The WM-BL group was characterized by increased abundance of *Burkholderia* sp., *Eubacterium*, and *Butyricimonas virosa*, while *Bacteroides fragilis* was specifically enriched in the HC group. These findings indicate that although no statistically significant genus-level differences were detected by traditional pairwise testing, LEfSe analysis revealed distinct microbial signatures associated with each group, highlighting its utility in identifying subtle yet biologically relevant variations in microbial composition.

To complement the differential abundance analyses above, pairwise genus-level comparisons were also performed using a less stringent threshold (unadjusted *p* < 0.05 and |log_2_FC| > 0.5, without FDR correction) to identify enriched genera between each pair of groups. This relaxed criterion was adopted because the FDR-corrected Manhattan plot analysis (Figure 6A) did not identify significant genera, likely due to the small sample sizes in certain subgroups. In the INT-BL versus INT-AT comparison, genera enriched in INT-BL included uncultured, *Acetanaerobacterium*, *Shuttleworthi*a, *Ruminococcus*, *Anaerostipe*s, and *Weissella*; no genera were significantly enriched in INT-AT. For INT-BL versus WM-BL, enriched genera in INT-BL were *Anaerostipes*, *Allisonella*, *Anaerofilum*, Saccharimonadaceae, Paraeggerthella, Oscillospiraceae, Shuttleworthia, uncultured, and *Romboutsia*, whereas only *Eubacterium siraeum* group was enriched in WM-BL. In INT-BL versus HC, INT-BL was characterized by enrichment of *Eubacterium*, unclassified *Lachnospiraceae*, uncultured, *Peptococcu*s, *Romboutsia*, *Anaerofilu*m, *Saccharimonadaceae*, *Acetanaerobacterium*, and *Oscillospiraceae*; no genera were enriched in HC. In INT-AT versus WM-BL, *Eubacterium* was enriched in INT-AT, while *Coprobacter*, *Megamonas*, and *Paraprevotella* were enriched in WM-BL. For INT-AT versus HC, INT-AT showed enrichment of *Collinsella*, *Romboutsia*, *Eubacterium*, unclassified *Lachnospiraceae*, and *Incertae Sedis*, whereas *Selenomonas* was enriched in HC. In WM-BL versus HC, enriched taxa in WM-BL included unclassified *Lachnospiraceae*, *Adlercreutzia*, *Eubacterium*, *Barnesiella*, *Intestinimonas*, and *Muribaculaceae*, while *Slackia* was enriched in HC. The corresponding Manhattan plots are shown in Supplementary Figure S1.

### 3.7 Random forest models highlight discriminatory OTUs between groups

**Figure 7:**
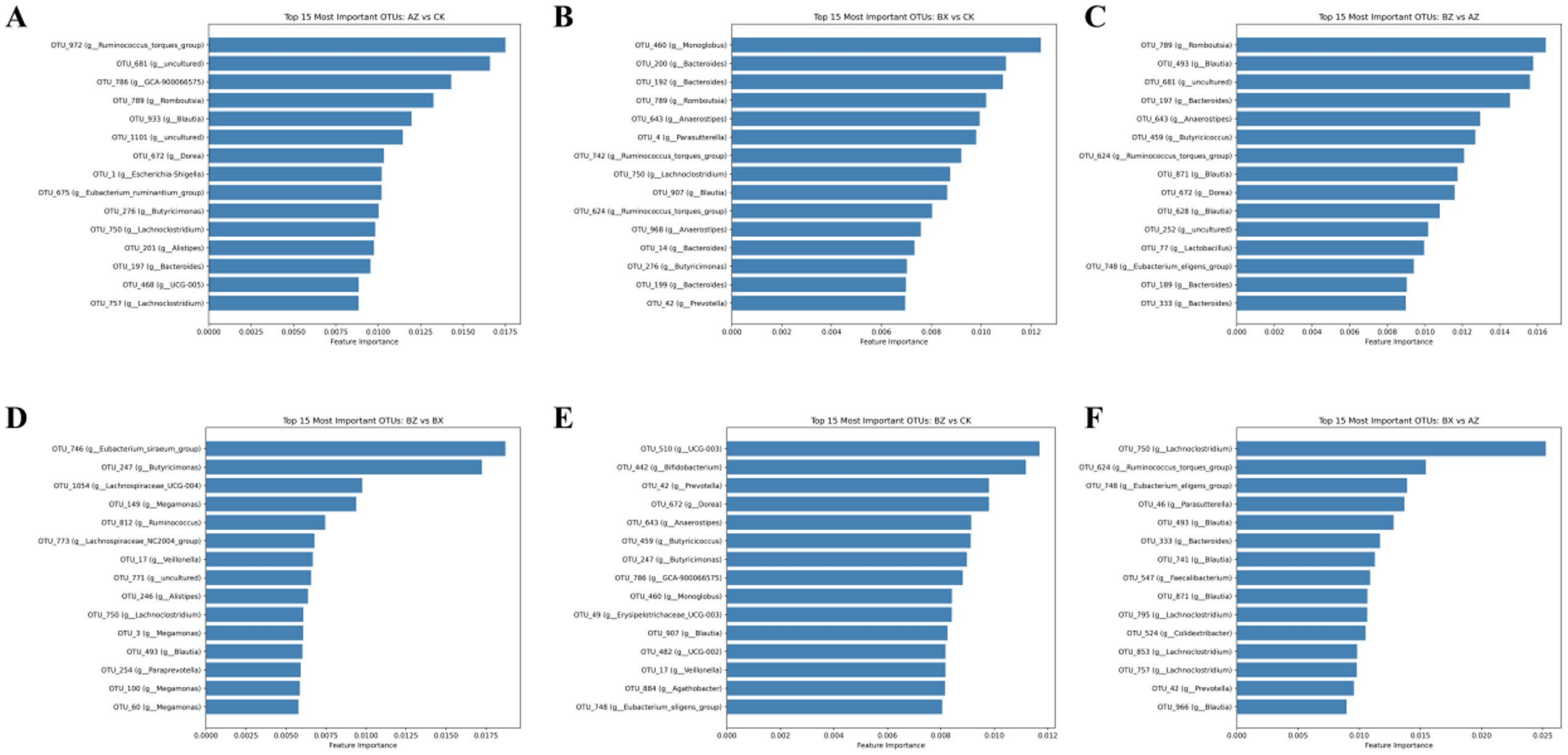
Top 15 OTUs contributing to group discrimination are shown for each pairwise comparison. **(A)** INT-BL (AZ) vs. HC (CK), **(B)** INT-AT (BX) vs. HC (CK), **(C)** WM-BL (BZ) vs. INT-BL (AZ), **(D)** WM-BL (BZ) vs. INT-AT (BX), **(E)** WM-BL (BZ) vs. HC (CK), **(F)** INT-AT (BX) vs. INT-BL (AZ). The x-axis represents feature importance (mean decrease in accuracy); the y-axis lists OTU IDs with genus-level annotation.

To identify discriminatory microbial features, pairwise random forest models were constructed between groups. For each comparison, the top 15 OTUs ranked by feature importance were selected, and their taxonomic annotation and scores are provided in Supplementary Tables S4–S9. In Figures 7A–F, groups are labeled using their sequencing identifiers: AZ (INT-BL), BX (INT-AT), BZ (WM-BL), and CK (HC). Throughout the text below, standardized abbreviations are used. In the INT-BL versus HC comparison (Figure 7A), OTU_832 (*Ruminococcus torques* group, *Lachnospiraceae*, *Firmicutes*) showed the highest importance, followed by OTU_681 (uncultured, *Lachnospiraceae*), OTU_786 (GCA-900066575), OTU_789 (*Romboutsia*), and OTU_833 (*Blautia*). Other notable features included OTU_1100 (uncultured), OTU_672 (*Dorea*), OTU_1 (*Escherichia-Shigella*), OTU_875 (*Eubacterium ruminantium* group), OTU_278 (*Butyricimonas*), OTU_750 (*Lachnoclostridium*), OTU_203 (*Alistipes*), OTU_397 (*Bacteroides*), OTU_468 (UCG-005), and OTU_757 (*Lachnoclostridium*). For INT-AT versus HC (Figure 7B), the leading feature was OTU_460 (*Monoglobus*), followed by OTU_200 and OTU_192 (both Bacteroides), OTU_789 (*Romboutsia*), and OTU_689 (*Anaerostipes*). OTUs from *Ruminococcus* and *Blautia* also contributed substantially. In WM-BL versus INT-BL (Figure 7C), OTU_789 (*Romboutsia*) had the highest importance (0.0164), followed by OTU_493 (*Blautia*), OTU_681 (uncultured Lachnospiraceae), OTU_197 (Bacteroides), and OTU_643 (*Anaerostipes*). Genera such as Butyricicoccus and *Dorea* were also identified. For WM-BL versus INT-AT (Figure 7D), OTU_746 (*Eubacterium siraeum* group) ranked first, with other top OTUs including OTU_247 (*Butyricimonas*), OTU_1054 (*Lachnoclostridium* UCG-004), OTU_149 (*Megamonas*), and OTU_812 (*Ruminococcus*). Additional discriminators included *Veillonella* and *Alistipes*. In the WM-BL versus HC comparison (Figure 7E), OTU_510 (UCG-003, Oscillospiraceae, Firmicutes) was most important, followed by OTU_442 (*Bifidobacterium*), OTU_42 (*Prevotella*), OTU_672 (*Dorea*), and OTU_643 (*Anaerostipes*). Other highlighted taxa included *Butyricicoccus*, unclassified genus in *Oscillospiraceae*, and *Veillonella*. Finally, in the INT-AT versus INT-BL comparison (Figure 7F), OTU_750 (*Lachnoclostridium*, *Lachnospiraceae*, *Firmicutes*) showed the highest importance, followed by OTU_624 (*Ruminococcus torques* group), OTU_748 (*Eubacterium siraeum* group), OTU_46 (*Parasutterella*), and OTU_493 (*Blautia*). Other notable features included OTU_333 (*Bacteroides*), OTU_741 (*Blautia*), OTU_347 (*Faecalibacterium*), OTU_871 (*Blautia*), OTU_795 (*Lachnoclostridium*), OTU_524 (*Clostridia*), OTU_853 (*Lachnoclostridium*), OTU_757 (*Lachnoclostridium*), OTU_42 (*Prevotella*), and OTU_960 (*Blautia*). Across all pairwise analyses, OTUs from families *Lachnospiraceae*, *Peptostreptococcaceae*, *Ruminococcaceae*, and *Bacteroidaceae*, as well as genera such as *Lachnoclostridium*, *Blautia*, *Anaerostipes*, *Faecalibacterium*, Eubacterium, *Prevotella*, and *Bacteroides*, were repeatedly identified. Most discriminatory OTUs belonged to the phyla Firmicutes and *Bacteroidota*, highlighting their central role in differentiating groups.

### 3.8 Random forest classification and ROC analysis of pairwise comparisons

**Figure 8:**
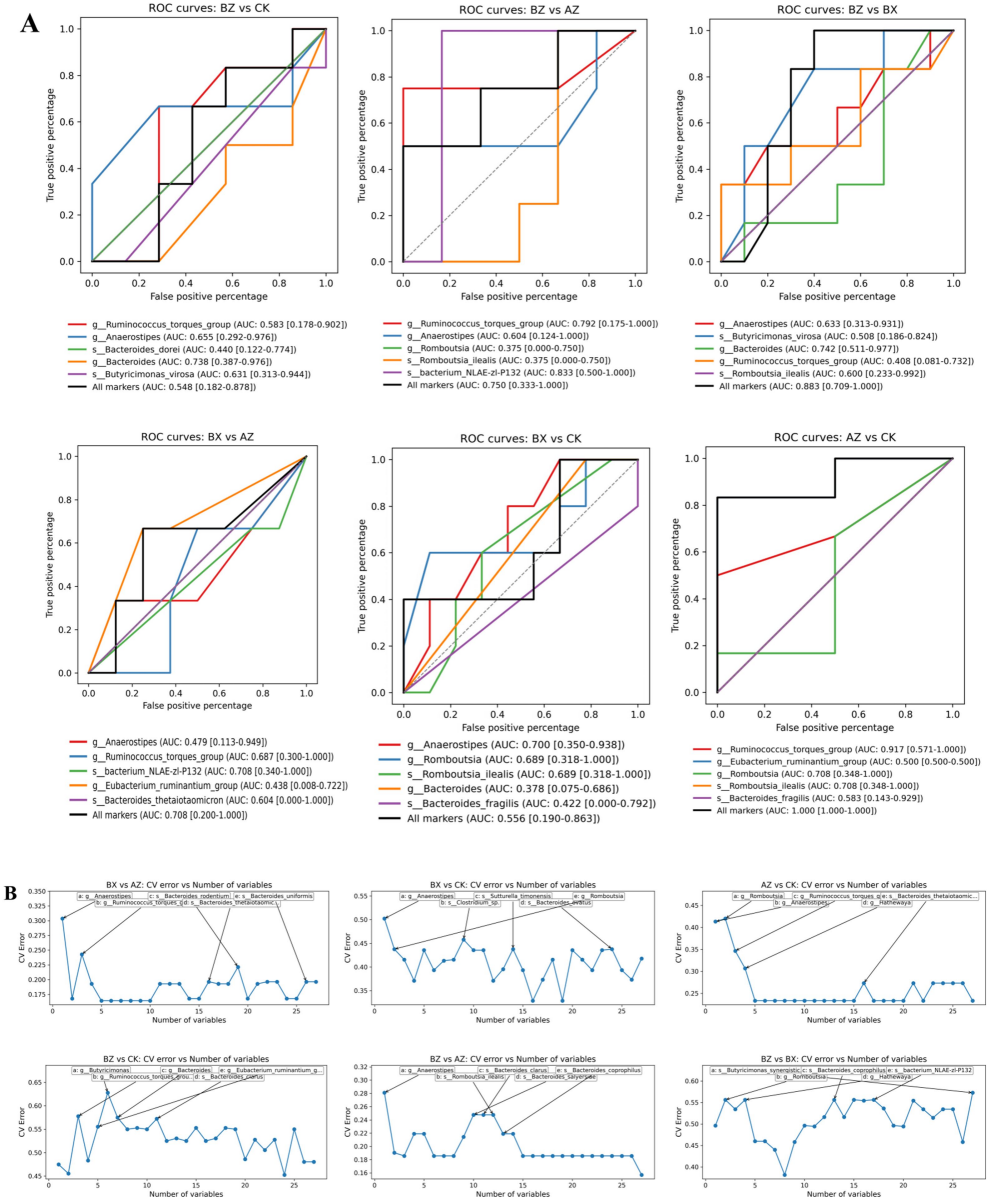
Random forest classification performance for pairwise group comparisons. **(A)** ROC curves showing the discriminatory performance of individual marker genera (colored lines) and the combined marker panel (black line) for each pairwise comparison. AUC values with 95% confidence intervals are shown in the legend. **(B)** Cross-validation (CV) error curves showing classification error as a function of the number of selected genera. The top 5 genera contributing to the model are annotated (a–e) for each comparison. The blue line connects CV error values at each variable number; the gray line represents the baseline error rate. Six pairwise comparisons are shown: BZ vs. CK (WM-BL vs. HC), BZ vs. AZ (WM-BL vs. INT-BL), BZ vs. BX (WM-BL vs. INT-AT), BX vs. AZ (INT-AT vs. INT-BL), BX vs. CK (INT-AT vs. HC), and AZ vs. CK (INT-BL vs. HC). Group labels follow sequencing identifiers as defined in Section 2.2.

To evaluate the discriminatory capacity of microbial markers, random forest classification models were constructed for all six pairwise comparisons, with model performance assessed by ROC analysis (Figure 8A) and optimal marker number determined by CV error curves (Figure 8B). Detailed AUC values for combined and individual markers are provided in Supplementary Tables S4–S9. Among all comparisons, INT-BL vs. HC (AZ vs. CK) achieved a combined AUC of 1.000 [1.000–1.000] under the 70/30 random split, with *Ruminococcus torques* group showing the highest individual discriminatory power (AUC = 0.917 [0.571–1.000]). However, the wide confidence intervals of individual markers (e.g., *Romboutsia*: 0.348–1.000; *Bacteroides fragilis*: 0.143–0.929) reflected the instability inherent in small-sample comparisons. To provide a more robust estimate, leave-one-out cross-validation (LOOCV) was performed using all available INT-BL and HC samples (*n* = 8 and *n* = 18, respectively), yielding a combined AUC of 0.865 [0.686–0.992] (Supplementary Figure S2). This confirms strong discriminability between baseline HSP patients and healthy controls while indicating that the perfect AUC under the 70/30 split likely reflected overfitting. Validation in an independent cohort is warranted. The WM-BL vs. INT-AT comparison (BZ vs. BX) also demonstrated strong separation (combined AUC = 0.883 [0.709–1.000]), driven primarily by *Bacteroides* (AUC = 0.742) and *Anaerostipes* (AUC = 0.633). In contrast, both INT-AT vs. HC (AUC = 0.556) and WM-BL vs. HC (AUC = 0.548) exhibited notably weaker combined performance, suggesting considerable microbial overlap between these groups and healthy controls. Importantly, the marked decline in discriminability from INT-BL vs. HC (LOOCV AUC = 0.865) to INT-AT vs. HC (AUC = 0.556) provides evidence that integrated treatment partially restored the gut microbiota toward a healthy-like profile. The other two comparisons, INT-AT vs. INT-BL (AUC = 0.708) and WM-BL vs. INT-BL (AUC = 0.750), showed intermediate performance (Figure 8A). CV error analysis consistently identified *Ruminococcus torques* group, *Anaerostipes*, *Bacteroides*, and *Romboutsia* among the top-ranked variables across multiple comparisons (Figure 8B), underscoring their potential as robust discriminatory taxa in HSP-associated dysbiosis.

### 3.9 Pairwise comparison of predicted gut microbial functional pathways among groups

**Figure 9:**
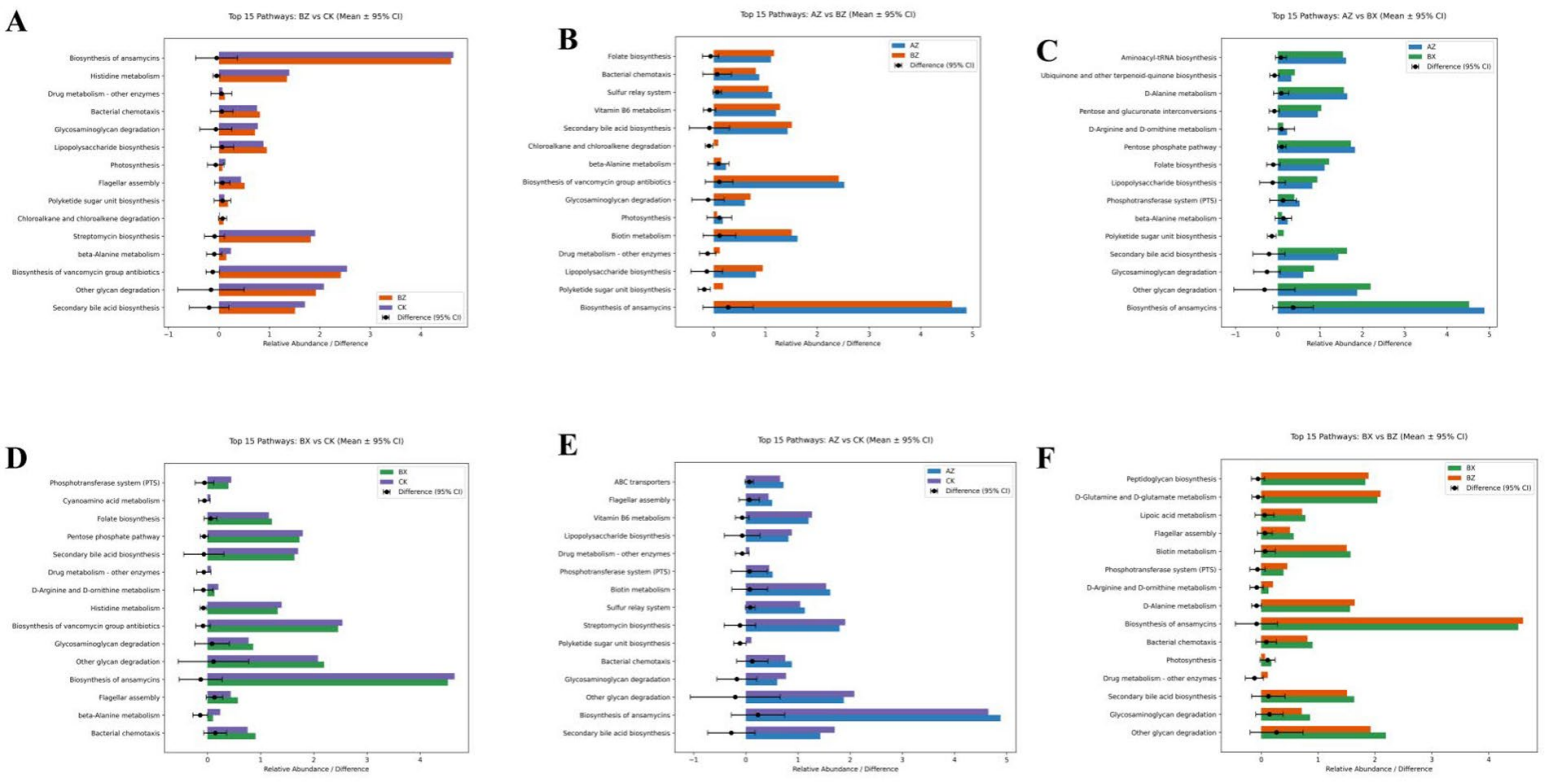
Differences in predicted KEGG pathways across groups. Pairwise comparisons of the top 15 most differentially abundant microbial pathways are shown: **(A)** INT-BL (AZ) vs. INT-AT (BX), **(B)** INT-BL (AZ) vs. WM-BL (BZ), **(C)** INT-BL (AZ) vs. HC (CK), **(D)** INT-AT (BX) vs. WM-BL (BZ), **(E)** INT-AT (BX) vs. HC (CK), **(F)** WM-BL (BZ) vs. HC (CK). Bar plots depict mean relative abundances and mean differences with 95% confidence intervals. Red asterisks indicate significance after FDR correction (FDR < 0.05). Functional annotations are based on KEGG and published literature.

To evaluate differences in the functional potential of gut microbiota, pairwise comparisons of predicted KEGG pathway abundances were conducted among the four groups using Student’s *t*-test with FDR correction. For each comparison, the top 15 pathways with the largest mean differences were visualized (Figures 9A–F). Overall, distinct functional trends were observed between groups. The INT-BL group showed higher relative abundance of pathways related to carbohydrate metabolism, such as glycolysis/gluconeogenesis and the citrate cycle, compared with INT-AT and HC. However, these differences did not remain statistically significant after FDR correction (FDR > 0.05). Among statistically significant findings, the polyketide sugar unit biosynthesis pathway showed significantly lower abundance in INT-BL than in INT-AT (mean difference [INT-BL minus INT-AT] = −0.1405, 95% CI: −0.2422 to −0.0388, FDR = 0.012), indicating that this pathway was upregulated following integrated treatment. Conversely, this pathway was significantly lower in INT-AT than in WM-BL (mean difference [INT-AT minus WM-BL] = −0.1812, 95% CI: −0.2981 to −0.0643, FDR = 0.0058), suggesting that despite the post-treatment increase, the integrated group remained below the WM baseline level. This pathway contributes to the formation of structural sugar units for polyketide antibiotics and other microbial secondary metabolites, which play roles in microbial competition and ecological fitness (Hertweck, 2009). In addition, the histidine metabolism pathway was significantly decreased in INT-AT compared with HC (mean difference = −0.0765, 95% CI: −0.1393 to −0.0137, FDR = 0.0215). This pathway is involved in nitrogen balance and the synthesis of bioactive molecules such as histamine, which can modulate immune responses and gut physiology (Smolinska et al., 2014). Other pathways, such as lipopolysaccharide biosynthesis, did not show significant differences after correction, but exhibited a trend toward lower abundance in the INT-BL group compared with INT-AT, suggesting a potential reduction in pro-inflammatory capacity. Similar non-significant trends were observed in WM-BL when compared to other groups, indicating that both integrated interventions may promote microbial functions related to energy metabolism and short-chain fatty acid production. Taken together, these findings indicate that, despite modest overall shifts in microbial function, specific pathways such as polyketide sugar unit biosynthesis and histidine metabolism were significantly altered. This suggests that the treatment may exert subtle but biologically relevant effects on the functional potential of the gut microbiota.

### 3.10 Co-occurrence network analysis reveals keystone taxa and ecological interactions

**Figure 10:**
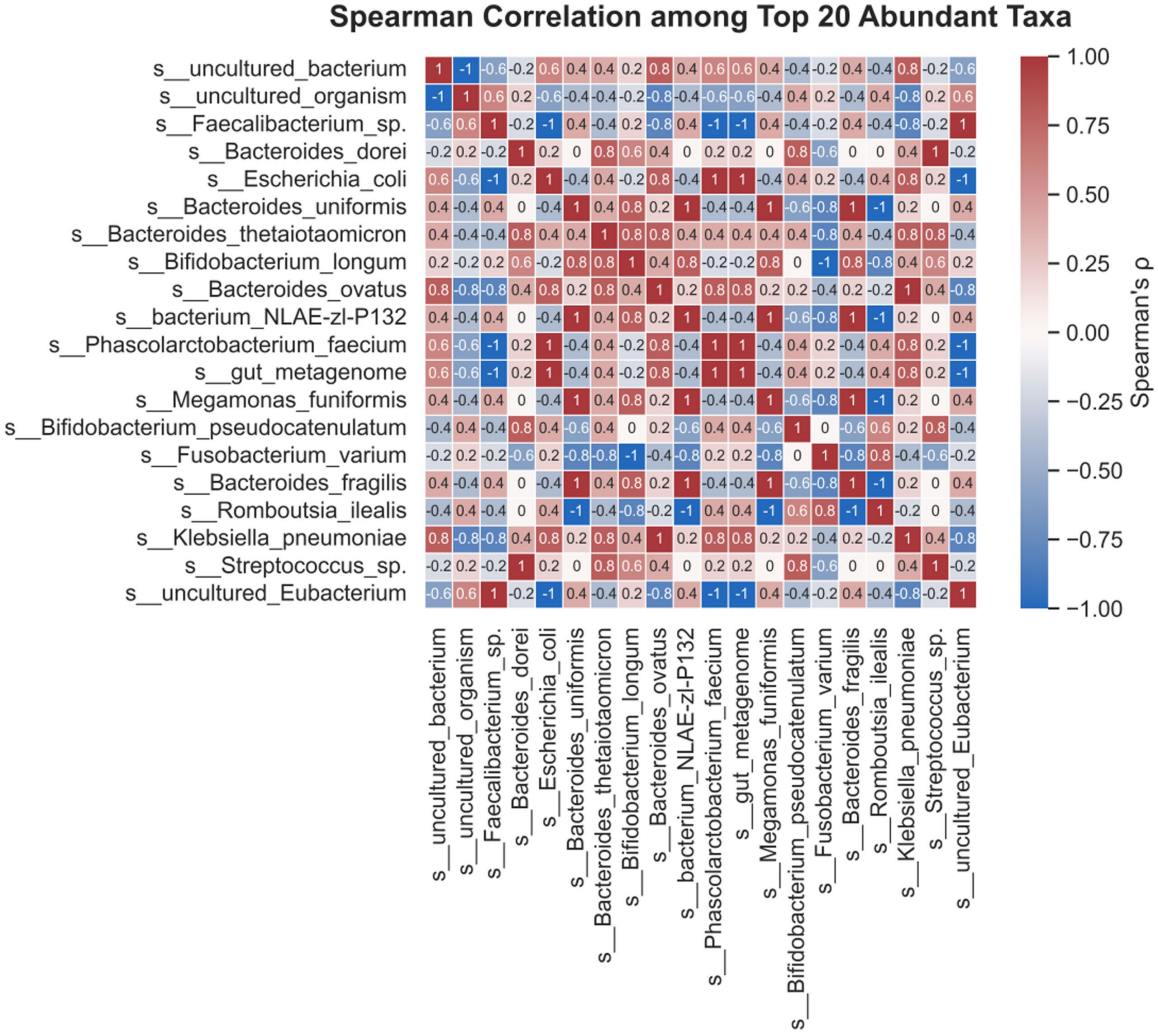
Microbial co-occurrence network based on Spearman correlation. Network constructed based on Spearman correlations among the top 20 most abundant taxa with adjusted *p* < 0.05 and |*ρ*| > 0.4. Nodes represent bacterial species; red edges indicate positive correlations, and blue edges indicate negative correlations. Node size reflects degree centrality. Network was constructed and visualized using Cytoscape v3.9.1.

To further explore the interaction network among these dominant taxa, a co-occurrence network was constructed based on significant Spearman correlations (adjusted *p* < 0.05, |*ρ*| > 0.4). In the resulting network (Figure 10), nodes represent microbial species and edges represent significant correlations, with red lines indicating positive associations and blue lines indicating negative associations. The network analysis revealed several highly connected taxa, such as *Bacteroides dorei*, which was positively linked to several other taxa. These patterns suggest the existence of key taxa that may act as ecological hubs or antagonists within the gut microbial community. Network topology parameters, such as node degree and betweenness centrality, further highlighted potential keystone species that may play important roles in maintaining gut microbial community structure and stability (Faust and Raes, 2012). The observed network of positive and negative correlations among dominant gut microbial taxa highlights the complexity of ecological interactions in the gut ecosystem. For example, *Bacteroides dorei* showed positive correlations with several taxa, suggesting potential cooperative interactions. Notably, a positive association was also observed between *Bacteroides dorei* and *Escherichia coli*; although *E. coli* is generally considered a pathobiont enriched in HSP patients (Section 3.5), such co-occurrence may reflect shared niche preferences rather than mutually beneficial interactions. Such intricate interaction networks are essential for the resilience, metabolic capacity, and homeostasis of the gut microbiota, and may ultimately influence host health outcomes (Coyte et al., 2015).

## 4 Discussion

HSP affects approximately 10–20 per 100,000 children annually worldwide, with growing recognition of the gut-immune axis’s role in disease pathogenesis and treatment response (Liang et al., 2024). At the molecular level, HSP is characterized by IgA1-dominant immune complex deposition in small vessels, triggering complement activation, neutrophil infiltration, and systemic vasculitis. These processes are closely linked to dysregulated immune responses and potential environmental triggers, such as infections and dietary antigens (Heineke et al., 2017). The gut microbiota plays a pivotal role in shaping immune development, regulating inflammation, and maintaining intestinal barrier integrity, thereby influencing systemic immunity through SCFA production and microbial metabolite signaling (Yoo et al., 2020). While previous studies have identified associations between microbial dysbiosis and immune-mediated or allergic diseases, most have focused on adult populations or animal models (Afzaal et al., 2022; Zheng et al., 2020). However, the pediatric immune system differs from that of adults, and the composition and dynamics of the pediatric gut microbiota are unique. Therefore, the specific role of gut microbiota in pediatric HSP pathogenesis and treatment response remains to be clarified.

In this study, participants were categorized as healthy controls, integrated TCM treatment group, and Western medicine treatment group based on treatment protocols and clinical outcomes (Figure 1). Using 16S rRNA gene sequencing, we investigated differences in gut microbiota composition and diversity, aiming to explore potential microbial mechanisms underlying therapeutic responses. Our results demonstrated marked alterations in microbial diversity and taxonomic composition between HSP patients and healthy controls. Notably, the integrated TCM treatment group exhibited restoration of beneficial bacteria following treatment, particularly Blautia and Faecalibacterium. In cross-sectional comparison, these genera were more abundant in the post-treatment integrated group (INT-AT) than in the WM baseline group (WM-BL), though longitudinal restoration in the WM group could not be assessed due to the absence of post-treatment samples. At baseline, potentially pathogenic taxa such as Escherichia-Shigella were elevated in both patient groups relative to HC. The restoration of these beneficial taxa coincided with clinical improvement, though formal correlation analysis between microbial abundance and clinical parameters was not performed in this study. These findings suggest that therapeutic modulation of the gut microbiota may be linked to treatment efficacy in pediatric HSP. However, given the observational and non-randomized nature of the study, treatment allocation was influenced by physician recommendation and guardian preference. Although baseline characteristics were comparable between groups, the potential for allocation bias should be considered when interpreting treatment effects.

Importantly, the study employed an add-on design in which both patient groups received an identical Western medicine regimen (vitamin C, ibuprofen, and loratadine as needed), with no corticosteroids, immunosuppressants, antibiotics, or probiotics administered in either group. The integrated group additionally received Compound Tuizi Decoction. Therefore, any between-group differences in microbiota outcomes can be primarily attributed to the herbal intervention. PERMANOVA analysis adjusting for age, sex, and baseline Shannon diversity confirmed that treatment group remained a significant explanatory factor for microbiota variation (*R*^2^ = 0.068, *p* = 0.023), further supporting the independent contribution of the herbal component. Nevertheless, we cannot completely exclude potential synergistic interactions between herbal and Western medicine components. Future studies employing a three-arm randomized design (TCM alone, WM alone, and integrated therapy) would provide stronger evidence for disentangling individual therapeutic contributions.

Specifically, our data demonstrated significant increases in *Blauti*a and *Faecalibacterium* in the integrated TCM group, which aligns with their recognized immunomodulatory roles (Luo et al., 2024; Zhang et al., 2022). *Blautia*, a key member of the *Lachnospiraceae* family, has been associated with anti-inflammatory effects and short-chain fatty acid production, and has been reported to be depleted in various immune-mediated and inflammatory conditions (Liu et al., 2021). Similarly, *Faecalibacterium* is a key butyrate-producing bacterium with strong anti-inflammatory effects and is associated with improved clinical outcomes across various immune-mediated diseases (Cabezas-Cruz and Bermudez-Humaran, 2024; Martin et al., 2023; Moon et al., 2023). In our cohort, the integrated group showed increased abundances of these beneficial bacteria after treatment, and cross-sectional comparisons indicated more favorable profiles than in the WM baseline group, suggesting that TCM interventions may exert broader effects on the gut-immune axis, potentially through multi-target actions of herbal compounds (Wang et al., 2024). In addition, *Lachnoclostridium* was identified as a key discriminatory taxon by random forest analysis. Although less well characterized, certain *Lachnoclostridium* species have been implicated in modulating mucosal immunity and influencing host metabolic pathways. The enrichment of these taxa following integrated treatment suggests a potential mechanistic link between microbiota modulation and immune regulation in pediatric vasculitis. While LEfSe analysis identified several taxa with significant differences between groups, it is important to note that these findings were not fully supported by conventional pairwise comparisons after FDR correction. This discrepancy reflects the methodological differences between the two approaches. LEfSe combines non-parametric statistical testing with LDA to detect features with both statistical significance and high effect size, but it does not implement multiple testing correction in the same manner as FDR-adjusted methods. As such, LEfSe may be more sensitive in detecting subtle group differences but also carries a higher risk of false positives. Therefore, the identified biomarkers should be interpreted as exploratory and hypothesis-generating, and further validation in larger cohorts or via metagenomic methods is warranted.

The differential microbiota responses observed across treatment groups highlight the heterogeneity of host–microbe interactions in pediatric HSP (Lozupone et al., 2012). To date, few studies have examined the relationship between HSP treatment modalities and gut microbial dynamics in children; most available data pertain to adult autoimmune diseases or evaluate single therapeutic strategies. Nevertheless, findings from related fields provide relevant literature. Chen et al. (2019) observed comparable reductions in alpha diversity and changes in beta diversity in a mouse model of gut dysbiosis under environmental stress. Similarly, studies by Shin et al. (2022) and Yang et al. (2021) demonstrated that interventions such as co-housing or probiotic treatment could modulate gut microbial diversity in aging and colorectal cancer models. These observations are in line with our results and highlight the value of microbial diversity indices as indicators of disease progression and therapeutic efficacy. Our findings suggest that integrated TCM therapy not only improves microbial diversity but also promotes the recovery of key beneficial taxa. This observation aligns with prior evidence that herbal medicines can act as prebiotics, selectively promoting the growth of beneficial bacteria while suppressing pathogenic species (Feng et al., 2018). Among the changes observed, an increase in butyrate-producing bacteria such as *Faecalibacterium* is particularly important, as butyrate contributes to anti-inflammatory and immune-regulatory processes (Siddiqui and Cresci, 2021). While clinical efficacy was generally comparable between treatment groups, the TCM group demonstrated more favorable microbiota profiles, suggesting that microbiota-mediated mechanisms may contribute to therapeutic benefit. Several herbal components in the oral formulation have been reported to regulate gut microbiota composition, enhance mucosal immune tolerance, and modulate inflammatory pathways such as IL-17 and TNF-α (Zhang et al., 2020; Zhang et al., 2024; Zhan et al., 2025). These immunomodulatory effects may contribute to reduced vascular inflammation in HSP patients treated with integrated TCM therapy, though no significant difference in short-term recurrence rates was observed between treatment groups in this study.

To further explore the biological implications of microbial changes, we performed functional predictions based on 16S rRNA gene profiles. Although both treatment groups showed trends toward enhanced pathways associated with carbohydrate metabolism and SCFA production, statistical significance after FDR correction was observed only in polyketide sugar unit biosynthesis and histidine metabolism. These results indicate that, while overall functional remodeling was limited, certain microbial pathways may be selectively influenced by treatment. However, it is important to note that PICRUSt2-based predictions are inferential and rely on reference genomes. They do not reflect actual gene expression or metabolite levels. Therefore, these results should be interpreted as hypothesis-generating and validated through metagenomic or metabolomic analyses in future studies.

In addition, our machine learning analysis identified *Blautia*, *Faecalibacterium*, and *Lachnoclostridium* as key markers distinguishing clinical response groups. ROC analysis demonstrated that these genera achieved moderate to excellent predictive accuracy, with AUC values ranging from 0.548 to 0.883 across pairwise comparisons (Figure 8A). The INT-BL vs. HC comparison yielded a combined AUC of 1.000 under the 70/30 split; however, LOOCV provided a more conservative estimate of 0.865 [0.686–0.992], suggesting strong but not perfect discriminability. Given the small sample size of the INT-BL subgroup, the LOOCV estimate is considered more reliable. Given the small sample size of the INT-BL subgroup, the LOOCV estimate is considered more reliable. External validation in a larger independent cohort would be necessary to confirm the discriminatory value of these markers. While the reduced discriminability of INT-AT vs. HC (AUC = 0.556) indirectly supports that integrated treatment shifted the microbiota toward a healthier profile, supporting their potential utility as biomarkers for therapeutic monitoring (Figure 8). In addition to compositional and diversity changes, our co-occurrence network analysis provided further ecological insights into the gut microbiota of pediatric HSP. By evaluating significant correlations among the top 20 most abundant taxa, we identified highly connected species, such as *Bacteroides dorei*, which acted as potential ecological hubs within the microbial community. Notably, both positive and negative associations were observed, reflecting intricate cooperative and competitive relationships (Figure 10). These ecological interactions may contribute to the maintenance of community stability and resilience, as well as prevent the overgrowth of potentially harmful taxa (Faust and Raes, 2012; Coyte et al., 2015). The identification of keystone taxa and their interaction networks is essential for understanding gut microbial homeostasis in health and disease. Disruption of these networks may compromise ecosystem stability and influence host immune responses. Our findings emphasize the importance of evaluating not only microbial composition and function, but also the structural organization of microbial communities when assessing the effects of therapeutic interventions in pediatric HSP. Collectively, our results suggest that integrated TCM treatment facilitates the restoration of beneficial gut microbes, supports microbial network stability, and modulates functional pathways relevant to immune regulation in children with HSP. Regarding treatment safety, serum creatinine levels increased significantly in both groups after the 4-week treatment period (integrated group: 46.2 ± 9.3 to 51.8 ± 8.7 μmol/L, *p* = 0.008; WM group: 47.6 ± 9.1 to 55.9 ± 9.5 μmol/L, *p* < 0.001), with a more pronounced increase observed in the WM group. However, all post-treatment values remained within the normal pediatric reference range (typically < 60–80 μmol/L depending on age and sex). This transient elevation may reflect physiological fluctuations associated with disease recovery, increased dietary protein intake during the convalescent phase, or the pharmacological effects of ibuprofen, which is known to transiently affect renal hemodynamics through prostaglandin inhibition. Importantly, no patient developed clinical signs of renal impairment during the study period, and BUN levels remained stable in both groups, suggesting that this change is unlikely to be clinically significant. Nevertheless, routine renal function monitoring during HSP treatment remains advisable given the known risk of HSP-associated nephritis. Several limitations of this study should be acknowledged. First, the study was observational and non-randomized, with treatment allocation based on clinical judgment and guardian preference. Although baseline characteristics were comparable between groups, residual allocation bias cannot be entirely excluded, and future randomized controlled trials are needed to confirm the observed effects. Second, formal quantitative dietary records were not collected. Although all HSP patients received identical standardized dietary guidance and healthy controls shared a similar cultural dietary background, dietary variation may have influenced microbiota outcomes. Future studies should incorporate validated dietary assessment tools and adjust for dietary covariates in statistical models. Third, the sample size was relatively small, particularly in the INT-BL subgroup (*n* = 8), limiting statistical power for certain pairwise comparisons. Larger, multi-center studies are warranted to validate these findings. In addition, post-treatment fecal samples were not available for the WM group, which precluded longitudinal within-group microbiota comparisons and direct assessment of treatment-induced microbial changes in this arm. Future studies should ensure complete longitudinal sampling across all treatment groups to enable more robust comparisons. Fourth, functional predictions were based on 16S rRNA gene sequencing and PICRUSt2 rather than whole-metagenome shotgun sequencing, which does not capture actual gene expression or metabolic activity. Integration of metagenomic, transcriptomic, and metabolomic approaches in future studies would provide more comprehensive mechanistic insights into gut microbiota–host interactions in pediatric HSP. Fifth, although the herbal decoction was prepared under standardized conditions with quality control procedures compliant with the Chinese Pharmacopeia, independent chromatographic fingerprinting and precise weight-based dosing were not implemented, limiting the ability to attribute specific microbiota changes to individual active compounds. Finally, the herbal formula was designed for the TCM syndrome of “blood-heat with stasis-toxin pattern”, and serum IgA levels were not systematically assessed, which limits the generalizability of findings to other syndrome patterns and the ability to fully characterize immunological mechanisms.

## 5 Conclusion

In conclusion, this study combined 16S rRNA gene sequencing with clinical data to investigate gut microbiota-related mechanisms underlying the therapeutic effects of integrated Chinese and Western medicine treatment in HSP. The integrated TCM group demonstrated a significant recovery of beneficial bacterial genera, particularly *Blautia* and *Faecalibacterium*. Although functional pathway analysis identified only a limited number of statistically significant alterations, a general trend toward metabolic restoration was observed. Furthermore, microbial co-occurrence network analysis revealed complex ecological interactions among dominant taxa, suggesting that both compositional and structural changes within the gut microbiota may contribute to immune modulation. Machine learning models additionally identified specific genera as potential microbial biomarkers for predicting treatment response.

These findings offer novel insights into the microbiota–immune interactions associated with integrated TCM therapy and support the potential role of microbiota modulation as a therapeutic mechanism. The add-on study design, in which both groups received identical Western medicine, provides a basis for attributing the incremental microbiota changes to the herbal intervention, although synergistic effects cannot be excluded. Nevertheless, short-term clinical outcomes were largely comparable between treatment groups, and the observational design of this study limits causal interpretation. Future research should focus on validating these findings in larger, randomized controlled trials with comprehensive dietary monitoring, chromatographic quality control of herbal formulations, and weight-based dosing protocols, and on exploring the therapeutic potential of microbiota-targeted interventions in the management of pediatric autoimmune diseases.

## Data Availability

The data presented in the study are deposited in the NCBI Sequence Read Archive (SRA) repository, accession number PRJNA1414245.
