# Supplementary material for: Clinical efficacy and gut microbiota profiling by 16S rRNA sequencing in children with Henoch–Schönlein purpura treated with integrated Chinese and Western medicine
Source: Front Microbiol. 2026 May 14;17:1697699. doi: 10.3389/fmicb.2026.1697699 (PMC13215996; doi:10.3389/fmicb.2026.1697699)
Supplement: Supplementary file 1 [file Supplementary_file_1.docx]

**Supplementary Material**

**Supplementary Figure**

**Supplementary Figure S1 Manhattan plots of differentially abundant genera across six pairwise comparisons.**

Each panel displays the significance of genus-level abundance differences as −log_10_ (*p*-value) on the y-axis, with genera arranged along the x-axis and grouped by phylum (color-coded). The horizontal dashed red line indicates the significance threshold of p = 0.05. Point size reflects significance level: the largest circles denote FDR < 0.01; medium circles denote unadjusted *p* < 0.01; smaller circles denote p < 0.05; and the smallest grey dots denote non-significant taxa (*p* ≥ 0.05). Phylum-level color assignments are shown in the upper-left legend of each panel. (A) INT-BL vs INT-AT (AZ vs BX); (B) INT-AT vs WM-BL (BX vs BZ); (C) INT-BL vs WM-BL (AZ vs BZ); (D) INT-AT vs HC (BX vs CK); (E) INT-BL vs HC (AZ vs CK); (F) WM-BL vs HC (BZ vs CK).

Group abbreviations: INT-BL, integrated treatment group at baseline; INT-AT, integrated treatment group after treatment; WM-BL, Western medicine group at baseline; HC, healthy controls. Sequencing identifiers (AZ, BX, BZ, CK) are as defined in Section 2.2.

**Supplementary Figure S2 Leave-one-out cross-validation (LOOCV) ROC curve for the random forest classification model distinguishing INT-BL (AZ, n = 8) from HC (CK, n = 18).**

The black solid line represents the LOOCV ROC curve based on the combined marker panel identified by random forest analysis. The gray dashed line indicates the performance of a random classifier (AUC = 0.5). The LOOCV AUC was 0.865 [95% CI: 0.686 - 0.992], with confidence intervals derived from 2,000 bootstrap resamples. This analysis was performed to provide a more robust estimate of classification accuracy given the small sample size of the INT-BL subgroup, as the 70/30 train-test split used in the primary analysis (Figure 8) yielded a perfect AUC of 1.000 that was likely inflated by overfitting. Group abbreviations: INT-BL, integrated treatment group at baseline; HC, healthy controls. Sequencing identifiers (AZ, CK) are as defined in Section 2.2.

Supplementary Figure S2 Fingerprint Profiling

Six batches of samples were analyzed according to the aforementioned sample and reference standard preparation methods, as well as the established chromatographic conditions. The resulting chromatograms were imported into the Similarity Evaluation System for Chromatographic Fingerprint of Traditional Chinese Medicine. Through chromatographic peak matching, 22 common characteristic peaks were determined. By comparing with the reference standards, 12 of these chromatographic peaks were successfully identified (the overlaid chromatograms and reference fingerprints are shown in Supplementary Figure 3). The similarities across all batches were greater than 0.9, demonstrating a high degree of consistency in the chemical composition among the different sample batches.


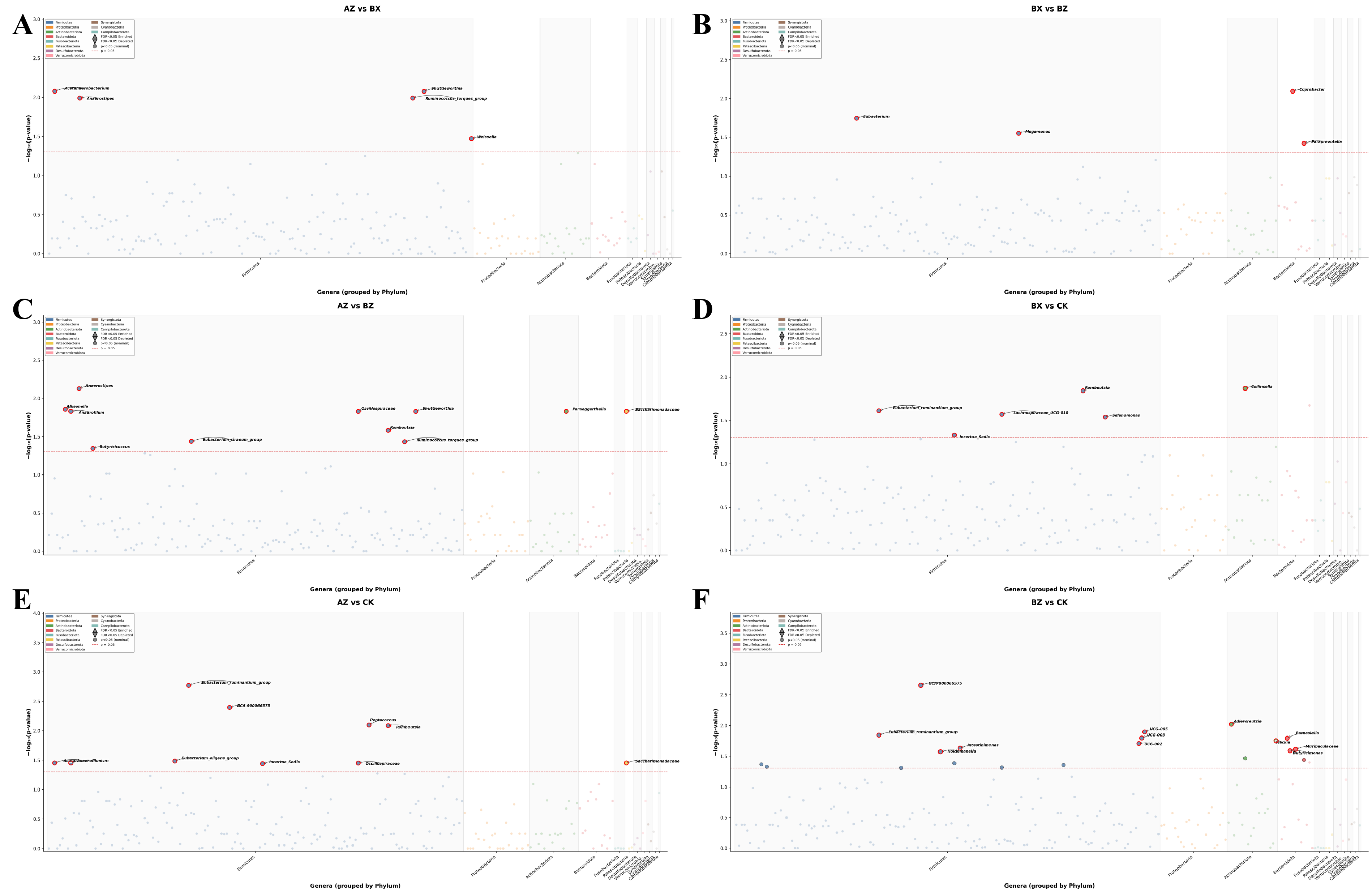


Supplementary Figure S1


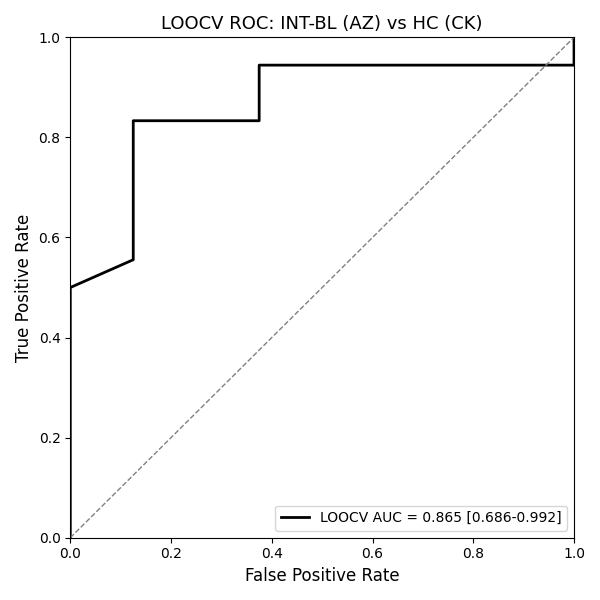


Supplementary Figure S2


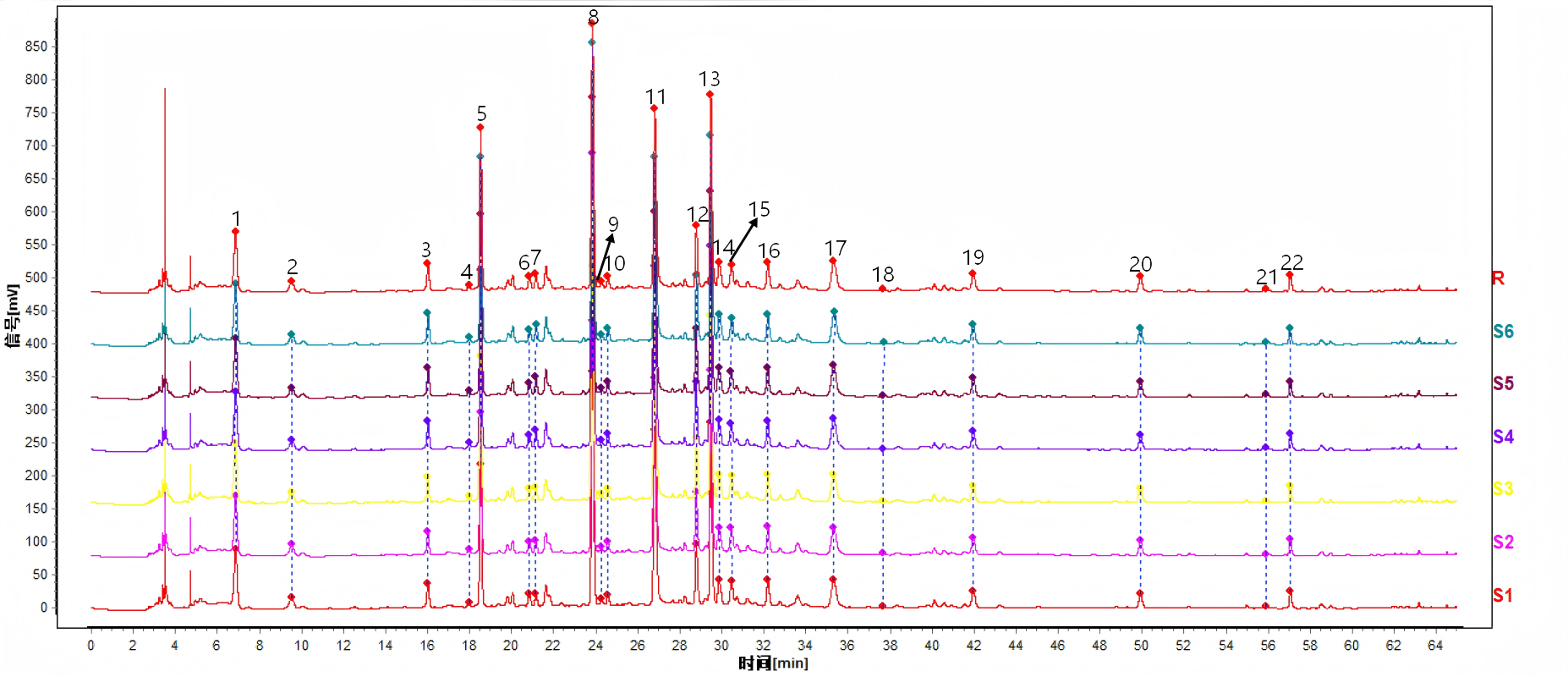


Supplementary Figure S3 HPLC fingerprint and reference fingerprint (R) of Compound Tuizi Decoction dry extract powder

Note: 2: Gallic acid; 4: N-acetyldopamine; 5: Loganic acid; 8: Gentiopicroside; 11: Paeoniflorin; 13: Forsythoside A; 15: Liquiritin; 16: Isoacteoside; 18: Rosmarinic acid; 19: Phillyrin; 21: Paeonol; 22: Glycyrrhizic acid.

**Supplementary Table Legends**

Table S1 Treatment Protocols for Integrated TCM and Western Medicine Groups

Supplementary Table S2a Quality Control Information for Compound Tuizi Decoction

Supplementary Table S2b Batch Information for Individual Decoction Pieces Used in Compound Tuizi Decoction

Supplementary Table S3 Body Weight Distribution and Estimated Weight-Adjusted Doses of Compound Tuizi Decoction

Table S4: Taxonomic annotation and feature importance of the top 15 OTUs identified by random forest analysis between the INT-BL (labeled AZ in sequencing data) and HC (labeled CK in sequencing data) groups.

Table S5 Taxonomic annotation and feature importance of the top 15 OTUs identified by random forest analysis between the INT-AT (labeled BX in sequencing data) and HC (labeled CK in sequencing data).

Table S6 Taxonomic annotation and feature importance of the top 15 OTUs identified by random forest analysis between the WM-BL (labeled BZ in sequencing data) and INT-BL (labeled AZ in sequencing data) groups.

Table S7 Taxonomic annotation and feature importance of the top 15 OTUs identified by random forest analysis between the WM-BL (labeled BZ in sequencing data) and INT-AT (labeled BX in sequencing data) groups.

Table S8 Taxonomic annotation and feature importance of the top 15 OTUs identified by random forest analysis between the WM-BL (labeled BZ in sequencing data) and HC (labeled CK in sequencing data) groups.

Table S9 Taxonomic annotation and feature importance of the top 15 OTUs identified by random forest analysis between the INT-AT (labeled BX in sequencing data) and INT-BL (labeled AZ in sequencing data) groups.

**Supplementary File S1 Inclusion and Exclusion Criteria**

This study involved pediatric participants and was conducted in accordance with ethical guidelines and approved by the Ethics Committee of Hubei Provincial Hospital of Traditional Chinese Medicine (Approval No. HBZY2022-C37-02).

1.1 Inclusion Criteria

Participants were enrolled if they met all of the following conditions:

① Age between 2 and 14 years

② Diagnosis of HSP based on the EULAR/PRINTO/PRES classification criteria

③ No prior administration of immunosuppressive therapy

④ Written informed consent obtained from a legal guardian

⑤ For the integrated group: clinical presentation consistent with the TCM syndrome pattern of “blood-heat with stasis-toxin”, as independently assessed by two senior TCM practitioners with more than 10 years of clinical experience, with discrepancies resolved by consensus

1.2 Exclusion Criteria

Participants were excluded if they met any of the following conditions:

① Renal impairment, defined as serum creatinine (Scr) > 80 µmol/L

② Use of antibiotics or probiotics within 4 weeks prior to sample collection

③ Known immunodeficiency or autoimmune diseases

④ History of gastrointestinal diseases, such as inflammatory bowel disease (IBD)

⑤ Incomplete data or failure to provide stool samples during follow-up

⑥ For the integrated group: clinical presentation predominantly characterized by “deficiency-cold” constitutional patterns, as determined by TCM syndrome differentiation

⑦ Significant deviation from the recommended hypoallergenic dietary regimen during the study period, as reported by guardians at follow-up visits

Supplementary Table S1 Treatment Protocols for Integrated TCM and Western Medicine Groups

| **No.** | **Type** | **Herbal Name (Latin & English)** | **Adult Dose (g)** | **Pediatric Dose (based on age)** | **Frequency** |
| --- | --- | --- | --- | --- | --- |
| 1 | Internal | Lithospermum erythrorhizon (Purple Gromwell) | 20 g | 1/3–1× adult dose | BID |
| 2 | Internal | Rehmannia glutinosa (Chinese Foxglove Root) | 12 g | 1/3–1× adult dose | BID |
| 3 | Internal | Paeonia rubra (Red Peony Root) | 12 g | 1/3–1× adult dose | BID |
| 4 | Internal | Moutan cortex (Tree Peony Bark) | 12 g | 1/3–1× adult dose | BID |
| 5 | Internal | Forsythia suspensa (Forsythia Fruit) | 10 g | 1/3–1× adult dose | BID |
| 6 | Internal | Achyranthes bidentata (Achyranthes Root) | 10 g | 1/3–1× adult dose | BID |
| 7 | Internal | Cicadae periostracum (Cicada Molting) | 8 g | 1/3–1× adult dose | BID |
| 8 | Internal | Gentiana macrophylla (Largeleaf Gentian) | 10 g | 1/3–1× adult dose | BID |
| 9 | Internal | Gypsum fibrosum (Gypsum) | 30 g | 1/3–1× adult dose | BID |
| 10 | Internal | Glycyrrhiza uralensis (Honey-fried Licorice) | 9 g | 1/3–1× adult dose | BID |
| — | External | No external herbal therapy applied | — | — | — |

Medicine Groups

A. Integrated Traditional Chinese Medicine (TCM) Group

Note: Pediatric doses were adjusted as follows:

Age 2–4 years: 1/3 adult dose

Age 4–6 years: 2/3 adult dose

Age ≥6 years: full adult dose

B. Western Medicine (WM) Group

| **No.** | **Drug Name** | **Dose** | **Frequency** | **Route** | **Indication** |
| --- | --- | --- | --- | --- | --- |
| 1 | Vitamin C | 100 mg/day | QD | Oral | Supportive therapy |
| 2 | Ibuprofen | 10 mg/kg/day (divided doses) | TID | Oral | Anti-inflammatory |
| 3 | Loratadine (PRN) | 5 mg | QD PRN | Oral | Allergic symptoms |

Supplementary Table S2a Quality Control Information for Compound Tuizi Decoction

| Item | Detail |
| --- | --- |
| Preparation type | Traditional water decoction |
| Preparation site | Traditional Chinese Medicine Pharmacy, Hubei Provincial Hospital of Traditional Chinese Medicine |
| Decoction equipment | Automated decoction machine |
| Standardized preparation parameters | soaking time: 30 min; first decoction: 30 min; second decoction: 20 min; final volume per bag: 150 mL |
| Source of decoction pieces | Hospital pharmacy stock, Hubei Provincial Hospital of Traditional Chinese Medicine |
| Authentication | All decoction pieces authenticated by licensed pharmacists at the Traditional Chinese Medicine Pharmacy in accordance with the Chinese Pharmacopoeia (2020 edition) |
| Contaminant testing | Performed by original suppliers; heavy metals (Pb, Cd, As, Hg, Cu), pesticide residues, and microbial contamination compliant with Chinese Pharmacopoeia 2020 limits |
| Compliance standard | Chinese Pharmacopoeia (2020 edition) |
| Batch information for individual herbs | Supplier names and batch numbers for each decoction piece are available upon request from the corresponding author |
| Independent HPLC/LC-MS fingerprinting | Currently underway; results expected within approximately one month and available for inclusion if the editorial timeline permits |

Supplementary Table S2b Batch Information for Individual Decoction Pieces Used in Compound Tuizi Decoction

| Herbal ingredient (Chinese name) | Herbal ingredient (Latin name) | Dose per adult formula (g) | Supplier | Batch number |
| --- | --- | --- | --- | --- |
| Zi Cao | Lithospermum erythrorhizon | 20 | Hubei Chenmei Chinese Medicine Co., Ltd | 2323030113 |
| Sheng Di Huang | Rehmannia glutinosa | 12 | Hubei Chenmei Chinese Medicine Co., Ltd | 2323060110 |
| Chi Shao | Paeonia rubra | 12 | Wuhan Hankou National Pharmaceutical Co., Ltd. | 202308008 |
| Mu Dan Pi | Moutan cortex | 12 | Hubei Tianji Pharmaceutical Co., Ltd. | 202308003 |
| Lian Qiao | Forsythia suspensa | 10 | Baohetang (Bozhou) Pharmaceutical Co., Ltd | C3061230051 |
| Niu Xi | Achyranthes bidentata | 10 | Hubei Chenmei Chinese Medicine Co., Ltd | 2323030118 |
| Chan Tui | Cicadae periostracum | 8 | Hubei Chenmei Chinese Medicine Co., Ltd | 2323030119 |
| Qin Jiao | Gentiana macrophylla | 10 | Hubei Tianji Pharmaceutical Co., Ltd | 202307027 |
| Sheng Shi Gao | Gypsum fibrosum | 30 | Hubei Tianji Pharmaceutical Co., Ltd | 202308021 |
| Zhi Gan Cao | Glycyrrhiza uralensis (honey-fried) | 9 | Hubei Chenmei Chinese Medicine Co., Ltd | 2322060302 |

Supplementary Table S3 Body Weight Distribution and Estimated Weight-Adjusted Doses of Compound Tuizi Decoction

| Age Group | n | Body Weight (kg, mean ± SD) | Dose Fraction | Estimated Total Daily Raw Herb Dose (g) | Estimated Dose (g/kg/day) |
| --- | --- | --- | --- | --- | --- |
| 2–4 years | 0 | 0 | 1/3 adult dose | 0 | 0 |
| 4–6 years | 0 | 0 | 2/3 adult dose | 0 | 0 |
| ≥6 years | 38 | 28.7 ± 5.6 | Full adult dose | 133.0 | 4.63 |

Note: Adult dose refers to the total weight of raw herbal decoction pieces per day (sum of all ingredients = 133 g raw herb equivalents). Estimated dose per kilogram was calculated as (total daily dose / mean body weight) for each age stratum. These estimates serve as a reference; individual variation exists within each age group. In the current cohort, all 38 enrolled patients in the integrated group were aged ≥6 years, and therefore all received the full adult dose. The 1/3 and 2/3 dose tiers were not utilized in this study. The full age-tiered dosing scheme is provided for completeness and reproducibility in future studies enrolling younger children.

**Table S4. Taxonomic annotation and feature importance of the top 15 OTUs identified by random forest analysis between BX and AZ groups.**

| #OTU ID | Feature Importance | Taxonomy |
| --- | --- | --- |
| OTU_789 | 0.016442431408088785 | k__Bacteria; p__Firmicutes; c__Clostridia; o__Peptostreptococcales-Tissierellales; f__Peptostreptococcaceae; g__Romboutsia; s__Romboutsia_ilealis |
| OTU_493 | 0.015774190207142044 | k__Bacteria; p__Firmicutes; c__Clostridia; o__Lachnospirales; f__Lachnospiraceae; g__Blautia; s__uncultured_bacterium |
| OTU_681 | 0.015600207241634717 | k__Bacteria; p__Firmicutes; c__Clostridia; o__Lachnospirales; f__Lachnospiraceae; g__uncultured; s__uncultured_bacterium |
| OTU_197 | 0.01453564536641165 | k__Bacteria; p__Bacteroidota; c__Bacteroidia; o__Bacteroidales; f__Bacteroidaceae; g__Bacteroides; s__uncultured_organism |
| OTU_643 | 0.012951744575793121 | k__Bacteria; p__Firmicutes; c__Clostridia; o__Lachnospirales; f__Lachnospiraceae; g__Anaerostipes; s__uncultured_organism |
| OTU_459 | 0.012696751586009898 | k__Bacteria; p__Firmicutes; c__Clostridia; o__Oscillospirales; f__Butyricicoccaceae; g__Butyricicoccus; s__Butyricicoccus_sp. |
| OTU_624 | 0.012098921243982827 | k__Bacteria; p__Firmicutes; c__Clostridia; o__Lachnospirales; f__Lachnospiraceae; g__Ruminococcus_torques_group; s__uncultured_bacterium |
| OTU_871 | 0.011755711516091753 | k__Bacteria; p__Firmicutes; c__Clostridia; o__Lachnospirales; f__Lachnospiraceae; g__Blautia; s__Blautia_sp. |
| OTU_672 | 0.01159086615603305 | k__Bacteria; p__Firmicutes; c__Clostridia; o__Lachnospirales; f__Lachnospiraceae; g__Dorea; s__uncultured_bacterium |
| OTU_628 | 0.010796188775216093 | k__Bacteria; p__Firmicutes; c__Clostridia; o__Lachnospirales; f__Lachnospiraceae; g__Blautia; s__uncultured_organism |
| OTU_252 | 0.010181809732355187 | k__Bacteria; p__Bacteroidota; c__Bacteroidia; o__Flavobacteriales; f__Flavobacteriaceae; g__uncultured; s__gut_metagenome |
| OTU_77 | 0.009967950071139714 | k__Bacteria; p__Firmicutes; c__Bacilli; o__Lactobacillales; f__Lactobacillaceae; g__Lactobacillus; s__Lactobacillus_fermentum |
| OTU_748 | 0.009426062542618375 | k__Bacteria; p__Firmicutes; c__Clostridia; o__Lachnospirales; f__Lachnospiraceae; g__Eubacterium_eligens_group; s__uncultured_organism |
| OTU_189 | 0.009046953359635495 | k__Bacteria; p__Bacteroidota; c__Bacteroidia; o__Bacteroidales; f__Bacteroidaceae; g__Bacteroides; s__bacterium_NLAE-zl-P132 |
| OTU_333 | 0.008989328099972806 | k__Bacteria; p__Bacteroidota; c__Bacteroidia; o__Bacteroidales; f__Bacteroidaceae; g__Bacteroides; s__uncultured_organism |

**Table S5. Taxonomic annotation and feature importance of the top 15 OTUs identified by random forest analysis between BX and CK groups.**

| #OTU ID | Feature Importance | Taxonomy |
| --- | --- | --- |
| OTU_460 | 0.012393559395145401 | k__Bacteria; p__Firmicutes; c__Clostridia; o__Monoglobales; f__Monoglobaceae; g__Monoglobus; s__uncultured_organism |
| OTU_200 | 0.010996245495808608 | k__Bacteria; p__Bacteroidota; c__Bacteroidia; o__Bacteroidales; f__Bacteroidaceae; g__Bacteroides; s__Bacteroides_fragilis |
| OTU_192 | 0.010874481811784053 | k__Bacteria; p__Bacteroidota; c__Bacteroidia; o__Bacteroidales; f__Bacteroidaceae; g__Bacteroides; s__Bacteroides_thetaiotaomicron |
| OTU_789 | 0.010198566952349468 | k__Bacteria; p__Firmicutes; c__Clostridia; o__Peptostreptococcales-Tissierellales; f__Peptostreptococcaceae; g__Romboutsia; s__Romboutsia_ilealis |
| OTU_643 | 0.00994334396926906 | k__Bacteria; p__Firmicutes; c__Clostridia; o__Lachnospirales; f__Lachnospiraceae; g__Anaerostipes; s__uncultured_organism |
| OTU_4 | 0.009798531466147037 | k__Bacteria; p__Proteobacteria; c__Gammaproteobacteria; o__Burkholderiales; f__Sutterellaceae; g__Parasutterella; s__uncultured_organism |
| OTU_742 | 0.00920061719944303 | k__Bacteria; p__Firmicutes; c__Clostridia; o__Lachnospirales; f__Lachnospiraceae; g__Ruminococcus_torques_group; s__uncultured_organism |
| OTU_750 | 0.008757676904611843 | k__Bacteria; p__Firmicutes; c__Clostridia; o__Lachnospirales; f__Lachnospiraceae; g__Lachnoclostridium; s__uncultured_organism |
| OTU_907 | 0.008640759803426788 | k__Bacteria; p__Firmicutes; c__Clostridia; o__Lachnospirales; f__Lachnospiraceae; g__Blautia; s__uncultured_bacterium |
| OTU_624 | 0.008034263832356081 | k__Bacteria; p__Firmicutes; c__Clostridia; o__Lachnospirales; f__Lachnospiraceae; g__Ruminococcus_torques_group; s__uncultured_bacterium |
| OTU_968 | 0.007579985984115244 | k__Bacteria; p__Firmicutes; c__Clostridia; o__Lachnospirales; f__Lachnospiraceae; g__Anaerostipes; s__uncultured_organism |
| OTU_14 | 0.007326274534670424 | k__Bacteria; p__Bacteroidota; c__Bacteroidia; o__Bacteroidales; f__Bacteroidaceae; g__Bacteroides; s__Bacteroides_dorei |
| OTU_276 | 0.007023205502158727 | k__Bacteria; p__Bacteroidota; c__Bacteroidia; o__Bacteroidales; f__Marinifilaceae; g__Butyricimonas; s__Butyricimonas_sp. |
| OTU_199 | 0.006980160159201237 | k__Bacteria; p__Bacteroidota; c__Bacteroidia; o__Bacteroidales; f__Bacteroidaceae; g__Bacteroides; s__Bacteroides_ovatus |
| OTU_42 | 0.006953199702353314 | k__Bacteria; p__Bacteroidota; c__Bacteroidia; o__Bacteroidales; f__Prevotellaceae; g__Prevotella; s__uncultured_bacterium |

**Table S6. Taxonomic annotation and feature importance of the top 15 OTUs identified by random forest analysis between BZ and AZ groups.**

| #OTU ID | Feature Importance | Taxonomy |
| --- | --- | --- |
| OTU_789 | 0.016442431408088785 | k__Bacteria; p__Firmicutes; c__Clostridia; o__Peptostreptococcales-Tissierellales; f__Peptostreptococcaceae; g__Romboutsia; s__Romboutsia_ilealis |
| OTU_493 | 0.015774190207142044 | k__Bacteria; p__Firmicutes; c__Clostridia; o__Lachnospirales; f__Lachnospiraceae; g__Blautia; s__uncultured_bacterium |
| OTU_681 | 0.015600207241634717 | k__Bacteria; p__Firmicutes; c__Clostridia; o__Lachnospirales; f__Lachnospiraceae; g__uncultured; s__uncultured_bacterium |
| OTU_197 | 0.01453564536641165 | k__Bacteria; p__Bacteroidota; c__Bacteroidia; o__Bacteroidales; f__Bacteroidaceae; g__Bacteroides; s__uncultured_organism |
| OTU_643 | 0.012951744575793121 | k__Bacteria; p__Firmicutes; c__Clostridia; o__Lachnospirales; f__Lachnospiraceae; g__Anaerostipes; s__uncultured_organism |
| OTU_459 | 0.012696751586009898 | k__Bacteria; p__Firmicutes; c__Clostridia; o__Oscillospirales; f__Butyricicoccaceae; g__Butyricicoccus; s__Butyricicoccus_sp. |
| OTU_624 | 0.012098921243982827 | k__Bacteria; p__Firmicutes; c__Clostridia; o__Lachnospirales; f__Lachnospiraceae; g__Ruminococcus_torques_group; s__uncultured_bacterium |
| OTU_871 | 0.011755711516091753 | k__Bacteria; p__Firmicutes; c__Clostridia; o__Lachnospirales; f__Lachnospiraceae; g__Blautia; s__Blautia_sp. |
| OTU_672 | 0.01159086615603305 | k__Bacteria; p__Firmicutes; c__Clostridia; o__Lachnospirales; f__Lachnospiraceae; g__Dorea; s__uncultured_bacterium |
| OTU_628 | 0.010796188775216093 | k__Bacteria; p__Firmicutes; c__Clostridia; o__Lachnospirales; f__Lachnospiraceae; g__Blautia; s__uncultured_organism |
| OTU_252 | 0.010181809732355187 | k__Bacteria; p__Bacteroidota; c__Bacteroidia; o__Flavobacteriales; f__Flavobacteriaceae; g__uncultured; s__gut_metagenome |
| OTU_77 | 0.009967950071139714 | k__Bacteria; p__Firmicutes; c__Bacilli; o__Lactobacillales; f__Lactobacillaceae; g__Lactobacillus; s__Lactobacillus_fermentum |
| OTU_748 | 0.009426062542618375 | k__Bacteria; p__Firmicutes; c__Clostridia; o__Lachnospirales; f__Lachnospiraceae; g__Eubacterium_eligens_group; s__uncultured_organism |
| OTU_189 | 0.009046953359635495 | k__Bacteria; p__Bacteroidota; c__Bacteroidia; o__Bacteroidales; f__Bacteroidaceae; g__Bacteroides; s__bacterium_NLAE-zl-P132 |
| OTU_333 | 0.008989328099972806 | k__Bacteria; p__Bacteroidota; c__Bacteroidia; o__Bacteroidales; f__Bacteroidaceae; g__Bacteroides; s__uncultured_organism |

**Table S7. Taxonomic annotation and feature importance of the top 15 OTUs identified by random forest analysis between BZ and BX groups.**

| #OTU ID | Feature Importance | Taxonomy |
| --- | --- | --- |
| OTU_746 | 0.018708713509645106 | k__Bacteria; p__Firmicutes; c__Clostridia; o__Oscillospirales; f__Ruminococcaceae; g__Eubacterium_siraeum_group; s__uncultured_bacterium |
| OTU_247 | 0.01723952988535611 | k__Bacteria; p__Bacteroidota; c__Bacteroidia; o__Bacteroidales; f__Marinifilaceae; g__Butyricimonas; s__Butyricimonas_virosa |
| OTU_1054 | 0.009759688300442016 | k__Bacteria; p__Firmicutes; c__Clostridia; o__Lachnospirales; f__Lachnospiraceae; g__Lachnospiraceae_UCG-004; s__uncultured_bacterium |
| OTU_149 | 0.00938768141141886 | k__Bacteria; p__Firmicutes; c__Negativicutes; o__Veillonellales-Selenomonadales; f__Selenomonadaceae; g__Megamonas; s__uncultured_organism |
| OTU_812 | 0.00745981301224318 | k__Bacteria; p__Firmicutes; c__Clostridia; o__Oscillospirales; f__Ruminococcaceae; g__Ruminococcus; s__uncultured_bacterium |
| OTU_773 | 0.006784300352396585 | k__Bacteria; p__Firmicutes; c__Clostridia; o__Lachnospirales; f__Lachnospiraceae; g__Lachnospiraceae_NC2004_group; s__uncultured_organism |
| OTU_17 | 0.006680259982523891 | k__Bacteria; p__Firmicutes; c__Negativicutes; o__Veillonellales-Selenomonadales; f__Veillonellaceae; g__Veillonella; s__uncultured_bacterium |
| OTU_771 | 0.006574771074001015 | k__Bacteria; p__Firmicutes; c__Clostridia; o__Lachnospirales; f__Lachnospiraceae; g__uncultured; s__uncultured_organism |
| OTU_246 | 0.0063761914997653765 | k__Bacteria; p__Bacteroidota; c__Bacteroidia; o__Bacteroidales; f__Rikenellaceae; g__Alistipes; s__Alistipes_sp. |
| OTU_750 | 0.006088650280340532 | k__Bacteria; p__Firmicutes; c__Clostridia; o__Lachnospirales; f__Lachnospiraceae; g__Lachnoclostridium; s__uncultured_organism |
| OTU_3 | 0.00607889491431366 | k__Bacteria; p__Firmicutes; c__Negativicutes; o__Veillonellales-Selenomonadales; f__Selenomonadaceae; g__Megamonas; s__uncultured_organism |
| OTU_493 | 0.006036829369671465 | k__Bacteria; p__Firmicutes; c__Clostridia; o__Lachnospirales; f__Lachnospiraceae; g__Blautia; s__uncultured_bacterium |
| OTU_254 | 0.00592123836779069 | k__Bacteria; p__Bacteroidota; c__Bacteroidia; o__Bacteroidales; f__Prevotellaceae; g__Paraprevotella; s__uncultured_bacterium |
| OTU_100 | 0.005865668773181166 | k__Bacteria; p__Firmicutes; c__Negativicutes; o__Veillonellales-Selenomonadales; f__Selenomonadaceae; g__Megamonas; s__uncultured_bacterium |
| OTU_60 | 0.005792329685149375 | k__Bacteria; p__Firmicutes; c__Negativicutes; o__Veillonellales-Selenomonadales; f__Selenomonadaceae; g__Megamonas; s__Megamonas_funiformis |

**Table S8. Taxonomic annotation and feature importance of the top 15 OTUs identified by random forest analysis between BZ and CK groups.**

| #OTU ID | Feature Importance | Taxonomy |
| --- | --- | --- |
| OTU_510 | 0.011695528663474989 | k__Bacteria; p__Firmicutes; c__Clostridia; o__Oscillospirales; f__Oscillospiraceae; g__UCG-003; s__uncultured_bacterium |
| OTU_442 | 0.011182295316872912 | k__Bacteria; p__Actinobacteriota; c__Actinobacteria; o__Bifidobacteriales; f__Bifidobacteriaceae; g__Bifidobacterium; s__Bifidobacterium_longum |
| OTU_42 | 0.00980231484729072 | k__Bacteria; p__Bacteroidota; c__Bacteroidia; o__Bacteroidales; f__Prevotellaceae; g__Prevotella; s__uncultured_bacterium |
| OTU_672 | 0.009802177368691972 | k__Bacteria; p__Firmicutes; c__Clostridia; o__Lachnospirales; f__Lachnospiraceae; g__Dorea; s__uncultured_bacterium |
| OTU_643 | 0.009139524578716727 | k__Bacteria; p__Firmicutes; c__Clostridia; o__Lachnospirales; f__Lachnospiraceae; g__Anaerostipes; s__uncultured_organism |
| OTU_459 | 0.009121041757374854 | k__Bacteria; p__Firmicutes; c__Clostridia; o__Oscillospirales; f__Butyricicoccaceae; g__Butyricicoccus; s__Butyricicoccus_sp. |
| OTU_247 | 0.00897673967521179 | k__Bacteria; p__Bacteroidota; c__Bacteroidia; o__Bacteroidales; f__Marinifilaceae; g__Butyricimonas; s__Butyricimonas_virosa |
| OTU_786 | 0.008822737642728732 | k__Bacteria; p__Firmicutes; c__Clostridia; o__Lachnospirales; f__Lachnospiraceae; g__GCA-900066575; s__uncultured_bacterium |
| OTU_460 | 0.00842075064782505 | k__Bacteria; p__Firmicutes; c__Clostridia; o__Monoglobales; f__Monoglobaceae; g__Monoglobus; s__uncultured_organism |
| OTU_49 | 0.008411053703174645 | k__Bacteria; p__Firmicutes; c__Bacilli; o__Erysipelotrichales; f__Erysipelatoclostridiaceae; g__Erysipelotrichaceae_UCG-003; s__human_gut |
| OTU_907 | 0.0082524071569855 | k__Bacteria; p__Firmicutes; c__Clostridia; o__Lachnospirales; f__Lachnospiraceae; g__Blautia; s__uncultured_bacterium |
| OTU_482 | 0.008179704308083133 | k__Bacteria; p__Firmicutes; c__Clostridia; o__Oscillospirales; f__Oscillospiraceae; g__UCG-002; s__uncultured_bacterium |
| OTU_17 | 0.008178519796570251 | k__Bacteria; p__Firmicutes; c__Negativicutes; o__Veillonellales-Selenomonadales; f__Veillonellaceae; g__Veillonella; s__uncultured_bacterium |
| OTU_884 | 0.008161342842354 | k__Bacteria; p__Firmicutes; c__Clostridia; o__Lachnospirales; f__Lachnospiraceae; g__Agathobacter; s__uncultured_bacterium |
| OTU_748 | 0.008054856366438504 | k__Bacteria; p__Firmicutes; c__Clostridia; o__Lachnospirales; f__Lachnospiraceae; g__Eubacterium_eligens_group; s__uncultured_organism |

**Table S9. Taxonomic annotation and feature importance of the top 15 OTUs identified by random forest analysis between BZ and BX groups.**

| #OTU ID | Feature Importance | Taxonomy |
| --- | --- | --- |
| OTU_746 | 0.018708713509645106 | k__Bacteria; p__Firmicutes; c__Clostridia; o__Oscillospirales; f__Ruminococcaceae; g__Eubacterium_siraeum_group; s__uncultured_bacterium |
| OTU_247 | 0.01723952988535611 | k__Bacteria; p__Bacteroidota; c__Bacteroidia; o__Bacteroidales; f__Marinifilaceae; g__Butyricimonas; s__Butyricimonas_virosa |
| OTU_1054 | 0.009759688300442016 | k__Bacteria; p__Firmicutes; c__Clostridia; o__Lachnospirales; f__Lachnospiraceae; g__Lachnospiraceae_UCG-004; s__uncultured_bacterium |
| OTU_149 | 0.00938768141141886 | k__Bacteria; p__Firmicutes; c__Negativicutes; o__Veillonellales-Selenomonadales; f__Selenomonadaceae; g__Megamonas; s__uncultured_organism |
| OTU_812 | 0.00745981301224318 | k__Bacteria; p__Firmicutes; c__Clostridia; o__Oscillospirales; f__Ruminococcaceae; g__Ruminococcus; s__uncultured_bacterium |
| OTU_773 | 0.006784300352396585 | k__Bacteria; p__Firmicutes; c__Clostridia; o__Lachnospirales; f__Lachnospiraceae; g__Lachnospiraceae_NC2004_group; s__uncultured_organism |
| OTU_17 | 0.006680259982523891 | k__Bacteria; p__Firmicutes; c__Negativicutes; o__Veillonellales-Selenomonadales; f__Veillonellaceae; g__Veillonella; s__uncultured_bacterium |
| OTU_771 | 0.006574771074001015 | k__Bacteria; p__Firmicutes; c__Clostridia; o__Lachnospirales; f__Lachnospiraceae; g__uncultured; s__uncultured_organism |
| OTU_246 | 0.0063761914997653765 | k__Bacteria; p__Bacteroidota; c__Bacteroidia; o__Bacteroidales; f__Rikenellaceae; g__Alistipes; s__Alistipes_sp. |
| OTU_750 | 0.006088650280340532 | k__Bacteria; p__Firmicutes; c__Clostridia; o__Lachnospirales; f__Lachnospiraceae; g__Lachnoclostridium; s__uncultured_organism |
| OTU_3 | 0.00607889491431366 | k__Bacteria; p__Firmicutes; c__Negativicutes; o__Veillonellales-Selenomonadales; f__Selenomonadaceae; g__Megamonas; s__uncultured_organism |
| OTU_493 | 0.006036829369671465 | k__Bacteria; p__Firmicutes; c__Clostridia; o__Lachnospirales; f__Lachnospiraceae; g__Blautia; s__uncultured_bacterium |
| OTU_254 | 0.00592123836779069 | k__Bacteria; p__Bacteroidota; c__Bacteroidia; o__Bacteroidales; f__Prevotellaceae; g__Paraprevotella; s__uncultured_bacterium |
| OTU_100 | 0.005865668773181166 | k__Bacteria; p__Firmicutes; c__Negativicutes; o__Veillonellales-Selenomonadales; f__Selenomonadaceae; g__Megamonas; s__uncultured_bacterium |
| OTU_60 | 0.005792329685149375 | k__Bacteria; p__Firmicutes; c__Negativicutes; o__Veillonellales-Selenomonadales; f__Selenomonadaceae; g__Megamonas; s__Megamonas_funiformis |
