# Supplementary material for: Clinical efficacy and gut microbiota profiling by 16S rRNA sequencing in children with Henoch–Schönlein purpura treated with integrated Chinese and Western medicine
Source: Front Microbiol. 2026 May 14;17:1697699. doi: 10.3389/fmicb.2026.1697699 (PMC13215996; doi:10.3389/fmicb.2026.1697699)
Supplement: Supplementary file 2 [file Supplementary_file_2.docx]

Supplementary File S1: Inclusion and Exclusion Criteria

This study involved pediatric participants and was conducted in accordance with ethical guidelines and approved by the Ethics Committee of Hubei Provincial Hospital of Traditional Chinese Medicine (Approval No. HBZY2022-C37-02).

1.1 Inclusion Criteria

① Participants were enrolled if they met all of the following conditions:

② Age between 2 and 14 years

③ Diagnosis of HSP based on the EULAR/PRINTO/PRES classification criteria

④ No prior administration of immunosuppressive therapy

⑤ Written informed consent obtained from a legal guardian

1.2 Exclusion Criteria

① Participants were excluded if they met any of the following conditions:

② =Renal impairment, defined as serum creatinine (Scr) > 80 µmol/L

③ Use of antibiotics or probiotics within 4 weeks prior to sample collection

④ Known immunodeficiency or autoimmune diseases

⑤ History of gastrointestinal diseases, such as inflammatory bowel disease (IBD)

⑥ Incomplete data or failure to provide stool samples during follow-up

Supplementary Table S1. Treatment Protocols for Integrated TCM and Western

| **No.** | **Type** | **Herbal Name (Latin & English)** | **Adult Dose (g)** | **Pediatric Dose (based on age)** | **Frequency** |
| --- | --- | --- | --- | --- | --- |
| 1 | Internal | Lithospermum erythrorhizon (Purple Gromwell) | 20 g | 1/3–1× adult dose | BID |
| 2 | Internal | Rehmannia glutinosa (Chinese Foxglove Root) | 12 g | 1/3–1× adult dose | BID |
| 3 | Internal | Paeonia rubra (Red Peony Root) | 12 g | 1/3–1× adult dose | BID |
| 4 | Internal | Moutan cortex (Tree Peony Bark) | 12 g | 1/3–1× adult dose | BID |
| 5 | Internal | Forsythia suspensa (Forsythia Fruit) | 10 g | 1/3–1× adult dose | BID |
| 6 | Internal | Achyranthes bidentata (Achyranthes Root) | 10 g | 1/3–1× adult dose | BID |
| 7 | Internal | Cicadae periostracum (Cicada Molting) | 8 g | 1/3–1× adult dose | BID |
| 8 | Internal | Gentiana macrophylla (Largeleaf Gentian) | 10 g | 1/3–1× adult dose | BID |
| 9 | Internal | Gypsum fibrosum (Gypsum) | 30 g | 1/3–1× adult dose | BID |
| 10 | Internal | Glycyrrhiza uralensis (Honey-fried Licorice) | 9 g | 1/3–1× adult dose | BID |
| — | External | No external herbal therapy applied | — | — | — |

Medicine Groups

A. Integrated Traditional Chinese Medicine (TCM) Group

Note: Pediatric doses were adjusted as follows:

Age 2–4 years: 1/3 adult dose

Age 4–6 years: 2/3 adult dose

Age ≥6 years: full adult dose

B. Western Medicine (WM) Group

| **No.** | **Drug Name** | **Dose** | **Frequency** | **Route** | **Indication** |
| --- | --- | --- | --- | --- | --- |
| 1 | Vitamin C | 100 mg/day | QD | Oral | Supportive therapy |
| 2 | Ibuprofen | 10 mg/kg/day (divided doses) | TID | Oral | Anti-inflammatory |
| 3 | Loratadine (PRN) | 5 mg | QD PRN | Oral | Allergic symptoms |
